# A New Phage Lysin Isolated from the Oral Microbiome Targeting *Streptococcus pneumoniae*

**DOI:** 10.3390/ph13120478

**Published:** 2020-12-19

**Authors:** Imme van der Kamp, Lorraine A. Draper, Muireann K. Smith, Colin Buttimer, R. Paul Ross, Colin Hill

**Affiliations:** 1 APC Microbiome Ireland, University College Cork, T12 YT20 Cork, Ireland; imme.vanderkamp@ucc.ie (I.v.d.K.); L.Draper@ucc.ie (L.A.D.); muireann.smith@ucc.ie (M.K.S.); colin.buttimer@ucc.ie (C.B.); P.ross@ucc.ie (R.P.R.); 2 School of Microbiology, University College Cork, T12 YN60 Cork, Ireland

**Keywords:** *Streptococcus pneumoniae*, bacteriophage, lysins, antimicrobial therapy, biofilms

## Abstract

*Streptococcus pneumoniae* is highly pathogenic and causes several mucosal and invasive infections. Due to the rising number of multidrug-resistant (MDR) strains of *S. pneumoniae*, new antimicrobials with alternative mechanisms of action are urgently needed. In this study, we identified two new Streptococcal phages from the oral microbiome, 23TH and SA01. Their lysins, 23TH_48 and SA01_53, were recombinantly expressed, characterized and tested for their lethality. SA01_53 was found to only lyse its host strain of *S. anginosus*, while 23TH_48 was found to possess a broader lytic activity beyond its host strain of *S. infantis*, with several *S. pneumoniae* isolates sensitive to its lytic activity. 23TH_48 at a concentration of five activity units per mL (U/mL) was found to reduce cell counts of *S. pneumoniae* DSM 24048 by 4 log_10_ colony forming units per mL (CFU/mL) within 1 h and effectively prevented and destroyed biofilms of *S. pneumoniae* R6 at concentrations of 228.8 ng/µL and 14.3 ng/µL, respectively. Given its high lytic activity, 23TH_48 could prove to be a promising candidate to help combat pneumococcal infections.

## 1. Introduction

*Streptococcus* spp. are found in the upper respiratory tract, the intestine, on the skin, and are the dominant species in the human oral cavity. While some streptococci can be beneficial for the oral environment by producing molecules that can inhibit harmful bacteria [1], some species are pathogenic. Infections range from mild throat infections to systemic and life-threatening diseases such as streptococcal pharyngitis, Scarlett fever, necrotizing fasciitis, and toxic shock syndrome. One of these highly pathogenic species is *Streptococcus pneumoniae*, which can cause pneumonia, otitis, meningitis, and sepsis [2]. Lower respiratory tract infections are among the most deadly infectious diseases, [3] with *S. pneumoniae* being the most frequent cause of severe pneumonia [4]. Invasive pneumococcal diseases can be prevented with vaccines targeting the capsular polysaccharides (CPs) of pneumococcal strains. Unfortunately, these multivalent vaccines are limited to between 10 to 13 of the 97 known CP types [5], leading to incomplete protection. Further drawbacks of vaccines include a rising prevalence of non-vaccine serotypes in both carriage and disease [6], as well as an increase in the prevalence of non-encapsulated pneumococci [7]. Moreover, the development of pneumococcal resistance to commonly used antibiotics, such as penicillins, macrolides, tetracyclines, and fluoroquinolones is causing worldwide concern [8].

In the global fight against multidrug-resistant (MDR) bacterial strains, the discovery of novel and effective therapies is urgently needed and bacteriophages (phages) and their encoded phage lysins are promising candidates due to their high specificity and lytic activity [9]. Phage lysins are encoded by both lysogenic and lytic phages and are highly evolved cell wall hydrolases that can selectively break down several different peptidoglycan bonds. These enzymes are typically expressed inside the cell at the final stages of lytic phage infection to facilitate the release of progeny phages. When applied extracellularly, these proteins can kill bacteria (especially Gram-positive bacteria) by specifically and effectively cleaving the bacterial cell wall, leading to osmotic lysis. Lysins are currently undergoing extensive study for their potential as antimicrobials against Gram-positive pathogens such as *Staphylococcus aureus* [10], *Streptococcus pyogenes* [11], and *Streptococcus agalactiae* [12] as well as Gram-negative bacteria such as *Pseudomonas aeruginosa* [13] and *Acinetobacter baumanii* [14].

Several lysins have been shown to effectively kill different human pathogenic strains of *S. pneumoniae* [15]. Structurally, these lysins have a modular architecture, consisting of a catalytic domain and a cell wall binding domain (CWBD). Interestingly, the majority of such lysins harbor a CWBD that specifically recognize choline residues in the teichoic acids of *S. pneumoniae* and some related bacteria. One exception is the Cpl-7 phage lysin (accession no. P19385) originating from *S. pneumoniae* phage Cpl-7 with a CWBD that allows it to recognize and kill a broader range of streptococcal pathogens (*S. pneumoniae, S. pyogenes, S. mitis, S. dysgalactiae*) and even *Enterococcus faecalis* [16,17]. Lysins with choline-binding domains (CBDs) active against *Streptococcus* include the pneumococcal major autolysin LytA (accession no. P06653), the phage lysins Cpl-1 (accession no. P15057) and Pal (accession no. O03979) originating from *S. pneumoniae* phages Cp-1 and Dp-1, respectively. Both Cpl-1 and Cpl-7 are 1,4-*N*-acetylmuramidases [16,18], whereas LytA and Pal are *N*-acetylmuramoyl-L-alanine amidases [19,20]. All described lysins targeting *S. pneumoniae* have been shown to eradicate biofilms of antibiotic susceptible and MDR strains effectively and were found to be effective in animal models (reviewed in [21]).

In this study, we describe two new phage lysins (23TH_48 and SA01_53) encoded by phages isolated from the oral microbiome (23TH and SA01). Lysins 23TH_48 and SA01_53 possess homology respectively to lysins Pal and Cpl-7 at the amino acid level sharing the same configuration of catalytic and CWBDs. Further investigations were performed to test their ability to kill *S. pneumoniae*.

## 2. Results

### 2.1. General Phage Characteristics, Genomic and Phylogenetic Analysis

**Figure 1:**
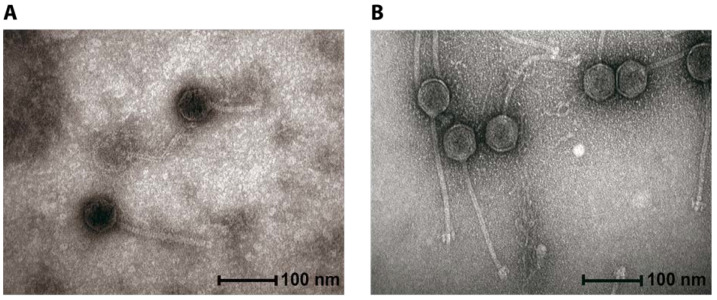
Transmission electron microscopy (TEM) images of *Streptococcus* phages 23TH (**A**) and SA01 (**B**).

Following extensive screening we can conclude that oral Streptococcal phages are not plentiful and those that we found were temperate in nature. We chose to characterize those that we did find and their lysins in this manuscript so that their existence and potential as therapeutics did not go unrecognized. Phages 23TH and SA01 were isolated from human saliva against the target strains *Streptococcus infantis* 23TH and *Streptococcus anginosus* SA01, respectively. These bacterial isolates were also obtained from the same saliva samples. The virions of both phages were found to possess a *Siphoviridae* morphology as evident by the long tail and the isometric capsids (Figure 1). The capsid of 23TH measures 55.9 nm ± 2.5 nm (*n* = 22), with a tail of 170.3 nm ± 8.1 nm (*n* = 9). The virion of SA01 possesses marginally different dimensions with a slightly longer tail of 190.6 nm ± 5.6 nm (*n* = 6) and a baseplate structure at the end of its tail. The head diameter of SA01 measured 62.2 nm ± 2.7 nm (*n* = 24).

**Figure 2:**
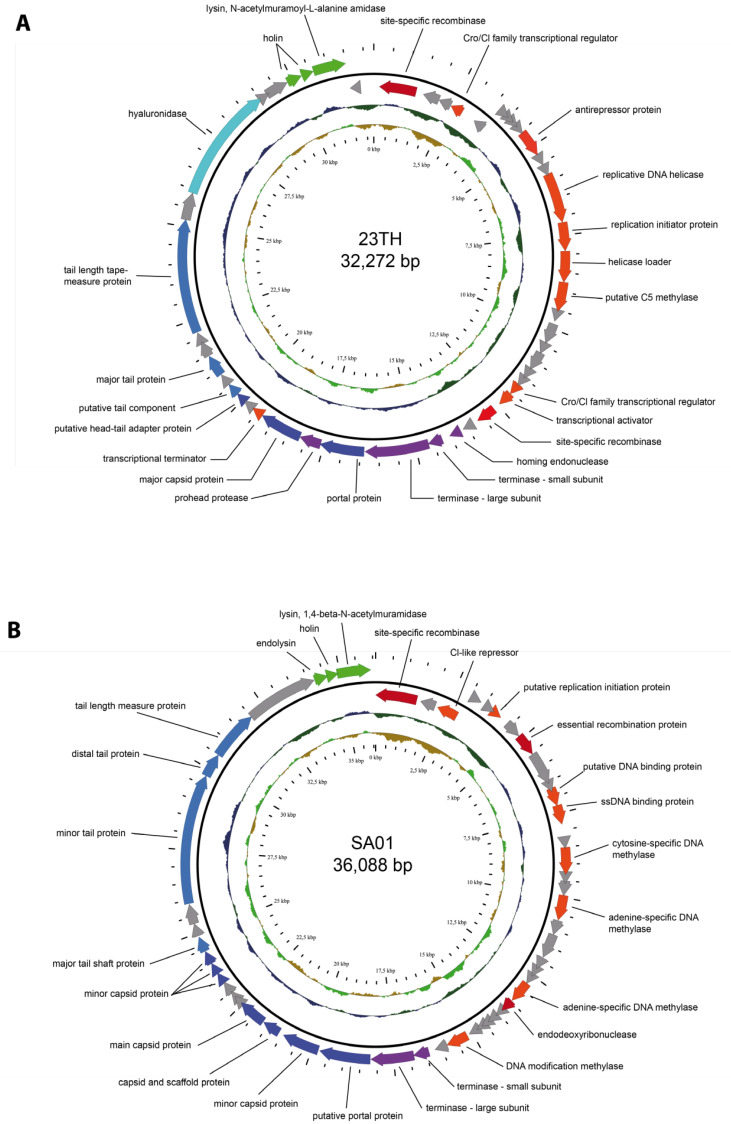
Annotated genomes of *Streptococcus* phages 23TH (**A**) and SA01 (**B**). On the outer ring, arrows represent ORFs: hypothetical proteins (grey), DNA replication, and regulation proteins (orange), proteins involved in recombination (red), packaging proteins (purple), phage structure proteins (blue), and lysis proteins (green). The middle ring represents GC content and the inner ring GC skew.

The genomes of both phages were sequenced (Figure 2). The genome of phage SA01 is 36,088 bp with a GC content of 37.54%. In total, 53 open reading frames (ORFs) were identified in its genome, of which three are found on the anti-sense strand (Figure 2B). Its genome possesses homology to other *Streptococcus* phages, with the closest match being *Streptococcus* phage Javan83 (Table S1, Figure S1A). Moreover, there are at least 29 phages on GenBank with shared identity ≥29% at the protein level (Geegenes employing the TBLASTX algorithm) (Figure S1B). No phage within this clade has been assigned taxonomy below the rank of *Siphoviridae*. These phages possess similar genome sizes, GC content, coding sequences (CDS), and tRNA gene number (Table S2). A gene encoding an integrase could be identified within the genomes of 20 of these 29 phages, indicating phages related to this cluster are capable of lysogeny. (Figure 2B). The typical GC content of these phages (c. 38%) is slightly less than commonly associated with the host species (*S. anginosus*) of SA01 at 39.0% (*n* = 74) [22].

The genome of *Streptococcus* phage 23TH is 32,272 bp in length with an overall GC content of 39.8%. Its genome contains 49 ORFs, of which 42 are read in the same direction (Figure 2A). Phage 23TH is evolutionarily distinct from phage SA01. Its closest relatives at the nucleotide level are *Streptococcus* phages Javan366, Javan363, and PH10, with a shared nucleotide identity ranging between 48% and 58% (Table S3, Figure S2A). At a protein level, there are at least 21 phages on GenBank identified with an identity of ≥29% to phage 23TH when using Geegenes employing the TBLASTX algorithm (Figure S2B). These phages all share similar genome sizes, GC content, CDS and tRNA gene number. No phage could be assigned a taxonomy below the rank of *Siphoviridae* (Table S4). Similar to phages related to SA01, an integrase encoding gene could be identified for 11 of these 24 phages indicating phages associated with this cluster are also capable of lysogeny. Moreover, several pneumococcal prophages (IPPX) [23] were found to share nucleotide identity (47–54%) with the 23TH phage (Figure S2B). The GC content of these phages (c. 39.7%) is similar to that associated with the host species of 23TH (*S. infantis*) at 39.3% (*n* = 11) [24]. 

Putative functions of the predicted ORFs were assigned based on a combination of BLASTP, InterProScan, HHpred, and the pVOG database. The functional ORFs were categorized into DNA replication and regulation proteins, phage virion structure (tail and capsid proteins), DNA packaging, lysis as well as proteins involved in recombination (Figure 2 and Supplementary Tables S5 and S6). For phage 23TH, putative functions were assigned to 25 of its 49 ORFs (51%), while for phage SA01 27 of 53 ORFs were annotated (51%). As mentioned genes encoding integrases and repressor proteins were found in the genomes of both phages suggesting they are temperate and capable of reproducing by either the lysogenic or lytic lifestyle. No tRNA or antibiotic resistance genes were identified. When performing BLAST (using the nr/nt database) on both phage genomes a significant number of hits to *Streptococcus* bacterial genomes were obtained, indicating relatives of these phages are highly adept at integrating into bacterial genomes.

The host range of phages 23TH and SA01 were tested using a panel of *Streptococcus* species by plaque assays but was found to be limited to their respective host strains *S. infantis* 23TH and *S. anginosus* SA01. Under the conditions used for plaque assays (see Section 4.2.), plaques of the SA01 phage were about 1 mm in diameter and hazy whereas plaques of the 23TH phage were clear, ranging between 1 and 3 mm in diameter. No plaques could be observed for 23TH phage on lawns of *S. bovis* i88, *S. dysgalactiae* UCC 5003, *S. infantarius* BAA-102, *S. mutans* SM1, *S. pyogenes* DSM2071, *S. salivarius* G85, *S. anguinis* CCUG 59327 and *S. uberis* U3. Unfortunately, we were unable to demonstrate if phage 23TH was capable of producing plaques on *S. pneumoniae* due to the difficulty with this species to form uniform bacterial lawns in overlay assays. Lawns typically retain pin-prick separations between colonies, which are not conducive to plaque visualization.

Wet lab experiments indicated that the efficiency of lysogeny is 58% for 23TH and 21% for SA01. Together with the presence of genes associated with lysogeny, both phages are likely to be unsuitable for therapeutic applications [25]. Therefore, we decided to look to the suitability of the endolysins encoded by these phages as antimicrobial agents rather than the phages themselves. 

### 2.2. Endolysins of Phages 23TH and SA01

Protein sequences were compared to previously characterized endolysins using BlastP, with domain assignment facilitated with InterproScan. Phylogenetic trees constructed with the top BlastP hits were further investigated (Figure S3). Interestingly, both lysins have similarities with phage lysins demonstrated to be active against *S. pneumoniae.* Lysin 23TH_48 has 77.97% identity (query cover: 100%) with lysin Pal of the phage Dp-1, whereas lysin SA01_53 has 76.32% identity (query cover: 100%) with lysin Cpl-7 lysin of phage Cp-7 (Figures S4 and S5).

**Figure 3:**
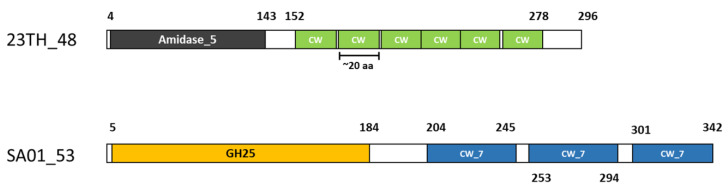
Lysin structures of 23TH_48 and SA01_53 as predicted with Pfam. Amidase 5 (*N*-acetylmuramoyl-L-alanine amidase, PF05382), CW (CW-binding-1, PF01473), Choline cell wall binding domain), GH25 (Glycoside hydrolase family 25 subfamily, PF01183) and CW_7 (CW_7 repeat, substrate binding domain, PF08230). Differences in domains are indicated by different colors.

Analysis of protein domains with Pfam revealed a two-domain structure for both lysins, comprising of an *N*-terminal catalytic domain and a C-terminal CWBD with a short linker sequence in between. The catalytic domain of lysin 23TH_48 is likely to be an *N*-acetylmuramoyl-L-alanine amidase (Amidase 5, PF05382), positioned next to six repeats of a cell wall/choline binding domain (CW, PF01473). Whereas, lysin SA01_53 harbors a 1,4- *N*-acetylmuramidase activity (GH25, PF01183), positioned alongside three repeats of a CW-7 substrate-binding domain (PF08230) (Figure 3).

### 2.3. Cloning and Expression of Lysins

Amplification of the lysin genes resulted in PCR products with lengths of 1203 bp (SA01_53) and 952 bp (23TH_48). Amplicons were cloned into pET-28b (+). After transformation and purification with His-tag chromatography, elution fractions were analyzed with SDS-PAGE to confirm the expression of soluble recombinant lysins. Expected protein bands were visible at ~42.0 kDa for SA01_53 and ~38.4 kDa for 23TH_48 (Figures S6 and S7).

### 2.4. Endolysin Host Range

The host range of these lysins was examined via spot assay on bacterial lawns of different strains (Table 1). SA01_53 was only active against *S. anginosus* SA01, the host strain of phage SA01. This finding is interesting considering that the SA01_53 lysin has homology to lysin Cpl-7, which has previously been shown to be highly active in killing *S. pneumoniae* and other Gram-positive strains in in vitro assays [19]. Lysin 23TH_48 was found to possess a broader lytic activity, being active against its phage host *S. infantis* 23TH as well as six other *S. pneumoniae* isolates (serotypes 9V (DSM 11865), 3 (DSM 14377) and 19F (DSM24048)) used in this study (Table S7), both in spot assays and turbidity reduction assays. No inhibition was observed against strains of other species of streptococci along with *L. lactis*, *S. aureus*, *E. faecalis* and *B. cereus* (see Table 1). As the SA01_53 lysin only showed activity on its own host strain and no inhibition of growth could be observed against other strains, we did not proceed with further investigations with this lysin. However, further study was carried out on lysin 23TH_48 due to its broad lytic activity against several *S. pneumoniae* strains. In several cases, it has been shown that the removal of the CWBD can improve the killing activity of a lysin [26,27,28]. However, such alterations can also lead to a loss of enzyme activity [29,30,31]. An investigation was conducted to determine whether truncated versions of the 23TH_48 lysin would result in a loss of activity as described for other proteins with CBDs [32]. Three truncated versions of 23TH_48 were created, using primers to create expression vectors harboring only the catalytic domains of the lysins or the catalytic domain plus one or more of the CWBDs (Tables S8 and S9). These truncated versions of 23TH_48 were expressed and were found to be soluble. However, none were found to be active on *S. infantis* or *S. pneumoniae* strains.

### 2.5. In Vitro Activity of 23TH_48

**Figure 4:**
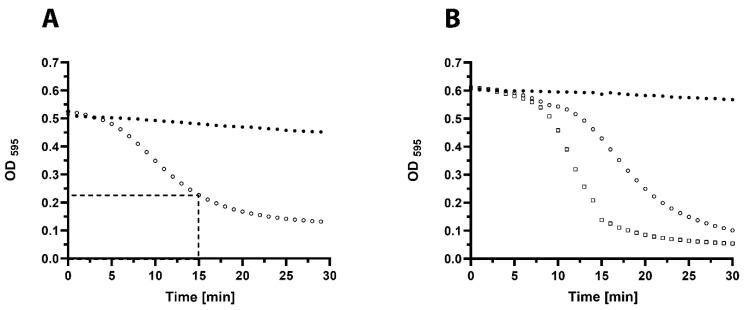
Turbidity reduction assays to determine 1 Unit (1U) of 23TH_48. Mid exponential cells were washed with phosphate buffered saline (PBS) and exposed to different concentrations of 23TH_48 at 37 °C to find the concentration that in 15 min halves the optical density (OD) at 595 nm. (**A**) *S. pneumoniae* R6 cells alone (filled circles), and in the presence of 1U of 23TH_48 which was found to be 64 ng/µL (open circles). (**B**) *S. pneumoniae* DSM 24048 cells (serotype 19F) alone (filled circles), and in the presence of 1U of 23TH_48 which was found to be between 1 ng/µL (open circles) and 1.5 ng/µL (open squares).

The in vitro activity of lysin 23TH_48 was further tested by turbidity reduction assays on *S. pneumoniae* strain R6 and serotype 19F strain DSM 24048. A common quantification method of antibacterial activity of purified lysins is to determine their capacity to decrease the turbidity of a suspension of bacterial cells over time. Here, we define 1 activity Unit (U) as the amount of enzyme which leads to a reduction of turbidity by 50% within 15 min at 37 °C in a suspension of bacterial cells harvested at mid-exponential growth, as shown in Figure 4.

Resultingly, 1U of 23TH_48 was determined to be 64 ng/µL against *S. pneumoniae* R6. For *S. pneumoniae* DSM 24048, 1U was equal to 1–1.5 ng/µL. Cell counts revealed that 1 U (5 activity units per mL (U/mL)) of 23TH_48 added to *S. pneumoniae* DSM 24048 led to a reduction of 4 log_10_ (CFU/mL) within 1 h. The addition of 2 U (10 U/mL) to the non-encapsulated *S. pneumoniae* R6 effectively reduced cell counts by 3.5 log_10_ colony forming units per mL (CFU/mL) (Figure S8).

### 2.6. In Vitro Biofilm Assays

*Streptococcus pneumoniae* often grows as a biofilm in many tissue infections, including recurrent middle-ear infections, otitis media with effusion, and chronic rhinosinusitis [33,34,35]. The use of antibiotics to remove biofilms is seen as critical [36]. The phage lysin Cpl-1 lysin has been shown to entirely prevent acute otitis media in mice colonized by *S. pneumoniae* [37]. Several lysins encoded by *S. pneumoniae* and its phages have also been tested successfully on in vitro biofilms [15,19]. Therefore, we investigated the ability of 23TH_48 to effectively kill planktonic cells before *S. pneumoniae* R6 biofilm formation, and also tested its ability to eradicate preformed biofilms using tetrazolium salt 2,3-bis[2-methyloxy-4-nitro-5-sulfophenyl]-2H-tetrazolium-5-carboxanilide (XTT) as a reporter for the presence of bacterial metabolic function and thus cell viability. 

**Figure 5:**
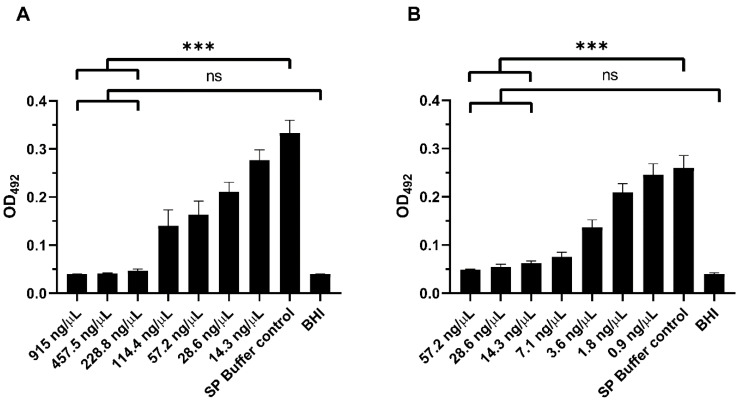
In vitro biofilm assays of *S. pneumoniae* R6 in BHI treated with 23TH_48 lysin in sodium phosphate buffer (SPB) measured with tetrazolium salt 2,3-bis[2 -methyloxy-4-nitro-5-sulfophenyl]-2H-tetrazolium-5-carboxanilide (XTT) assay at OD_492_. As a control, cells were incubated in BHI with the addition of SPB instead of lysin. (**A**) Prevention of biofilm formation by 23TH_48 in a concentration-dependent manner. (**B**) Treatment of biofilms by 23TH_48 in a concentration dependent manner. Statistical analysis with one-way ANOVA followed by Dunnet’s post-test showed no significant difference for the three highest lysin concentrations used when compared to the BHI control. These concentrations, however, all showed significance when compared with the untreated SP buffer control (*** *p*-value < 0.0001).

Lysin 23TH_48 was found to prevent biofilm formation by *S. pneumoniae*, at a concentration of 228.8 ng/µL, as this concentration of lysin reduced the measured XTT absorption (OD_492nm_) to that observed for the brain heart infusion (BHI) control (*p* = 0.9864), indicating effective prevention of biofilm formation in vitro (Figure 5A). A concentration of 57.2 ng/µL reduced the absorption by half with comparison to the control group that did not receive added lysin (SP buffer control vs. 57.2 ng/µL 23TH_48). A concentration-dependent reduction in the OD_492nm_ measurements was also observed for the destruction of established *S. pneumoniae* R6 biofilms (Figure 5B). Even though absorbance values for high lysin concentrations appeared elevated compared to the BHI negative control, mean differences were not statistically significant for lysin concentrations above 14.3 ng/µL. The XTT assay used here gives valuable information on the viability of cells in the biofilm revealing that the addition of ≥14.3 ng/µL of 23TH_48 reduced biofilm metabolic function to that observed for BHI media alone (BHI control vs. 14.3 ng/µL 23TH_48; *P* ≥ 0.0539). Biofilms were also significantly reduced compared to the untreated control (SP buffer control vs. 14.3 ng/µL 23TH_48; *p* < 0.0001). Similar results have been described for the lysin Pal on preformed biofilms with loss of cell viability confirmed using cell counts [16].

## 3. Discussion

Within this study, we describe the characterization of *Streptoccocus* phages 23TH and SA01 infecting *S. infantis* and *S. anginosus,* respectively. These phages were found to possess highly limited host ranges (limited to host strain) when assessed by plaque assays, with the examination of their genomes showing the presence of genes encoding proteins (integrases, repressor proteins) associated with a lysogenic lifestyle. Both phages were found to possess homology at the protein level (identity ≥29%, Geegenes employing the TBLASTX algorithm) to distinct clusters of related *Streptococcus* phages on Genbank. Phages within these related clusters were found to possess similar genomic properties (genome size, GC content, and CDS number), with both phages positioned in the family of *Siphoviridae* in no defined subfamily or genus. Historically, phage taxonomy has been based on phage virion morphology and nucleic acid composition. However, in recent years there has been a shift toward genome organization-based taxonomy that takes into consideration phage nucleotide, protein, and proteome homology. This shift has resulted in the extraction of phage genera and species from the families *Myoviridae* and *Podoviridae* to new families (*Autographiviridae* and *Herelleviridae,* for example) that better reflect their shared properties and evolutionary history [38]. Based on the brief phylogenetic analysis performed in this study with phages 23TH and SA01, it is clear that there is a genetic diversity among “*Siphoviridae*” phages that is not adequality described by current ICTV defined phage taxonomy.

Several phages within phage clusters related to 23TH and SA01 were found to possess genes encoding an integrase. Furthermore, BLASTN analysis of phages 23TH and SA01 using the nr/nt database showed several hits against streptococci genomes, indicating relatives of these phages form prophage elements within bacterial genomes. The former two points suggest that both 23TH and SA01 have evolved from phages where the lysogenic cycle comprises an important strategy for their continuity. Based on the discussed findings, such phages are unlikely to be worthwhile candidates for phage therapy. Therefore, an examination of the cell wall degrading proteins of phages 23TH and SA01 was conducted to assess their potential as antimicrobial agents.

Lysin SA01_53 (a 1,4-*N*-acetylmuramidase with CW_7 cell-binding domains), derived from phage SA01, was found to possess a narrow host range with lytic activity being limited to its host strain when assessed by spot assay on bacterial lawns. This was surprising given the fact that this lysin is closely related to lysin Cpl-7 (BLASTP: 100% coverage, 76% identity), derived from *S. pneumoniae* phage Cp-7. The cell-wall binding module in Cpl-7 is made of three identical repeats (CW_7 domains) which are structurally and sequentially unrelated to the choline-binding motifs typically associated with other pneumococcal phage lysins. It has been suggested that this lysin may possess a broad activity as its cell wall binding domain does not restrict its activity to strains containing choline-containing cell walls [39]. However, SA01_53 does not appear to have the same broad host range reported for Cpl-7, which shows lytic activity against *S. pneumoniae* and other Gram-positive strains when tested in in vitro assays [19]. One major difference was found between these two lysins, within the linker sequence (positions 184–204), which might explain why SA01_53 has a reduced host-range compared to Cpl-7. Linker sequences are gaining more attention recently, due to insight into their role in domain orientation and dynamics. For example, alterations in the linker sequence of the endolysin of mycobacteriophage D29 were shown to change the activity and specificity of the endolysin [40]. Alterations in the length and nature of the linker sequence were also found to be important for the function of endolysin Ply500, with removal of the linker sequence actually leading to loss of its activity [41]. Thus, we propose that the differences in the linker sequence seen in SA01_53 when compared to Cpl-7 could result in its narrow spectrum of activity. Alternatively, as can be the case with respect to the heterologous expression of proteins and their purification, there may have been issues with, for example, protein folding, temperature stability, or his-tag interference that reduced the full antimicrobial potential of this lysin [42].

Lysin 23TH_48 (a *N*-acetylmuramoyl-L-alanine amidase with CW cell-binding domains), derived from phage 23TH, was found to be active on its host strain *S. infantis* and several isolates of *S. pneumoniae* when tested similarly. The closest homolog of this protein is lysin Pal (BLASTP: 100% coverage, 78% identity) derived from *S. pneumoniae* phage Dp-1. This protein has also been reported to possess a broad lytic spectrum and was found to be active on several serotypes of *S. pneumoniae*, as well as on *S. oralis* and *S. mitis.* Interestingly, 10-fold concentrations of Pal were needed to show even minor reductions in cell counts of *S. oralis* and *S. mitis* compared to *S. pneumoniae*.[20]. Similar activity was observed for lysin Cpl-1*; i*n vitro experiments showed specific killing activity on *S. pneumoniae, S. oralis* and *S. mitis*. This spectrum of killing activity may be explained by the fact that these two species contain choline residues in the cell wall, which are very similar to those of *S. pneumoniae* [18]. In a recent paper, Pimenta et al. [43] showed that *S. infantis* is genetically related to *S. pneumoniae*. The similarities between these strains and the CWBD of lysins 23TH_48, Pal and Cpl-1 (Interpro: IPR018337) may explain why 23TH_48 has a similar specificity as Cpl-1 and Pal.

In this study, we created truncated versions of lysin 23TH_48, harboring only the catalytic domain of the lysin or the catalytic domain plus one or more of the CWBDs. However, enzymatic activity for these lysins was not retained with these subtractions. Such results indicate that the CWBD (a CW domain) of 23TH_48, which is similar to those of the autolysin LytA and pneumococcal phage lysins Pal and Cpl-1, is crucial for its killing activity. In general, CWBDs influence substrate specificity, the overall endolysin structure and cell wall binding affinity. Therefore, they are a major factor in contributing to the lethality of phage lysins. The typical choline-binding modules of the autolysin LytA and the Pal lysin are responsible for binding to choline residues in teichoic acid, typically found in pneumococcal cell walls. It has been shown that the folding and stability of LytA is directly linked to the choline-binding mechanism, which promotes the dimerization of the protein. Therefore, the CBDs are required for optimal substrate recognition and the activity of the lysins [32]. Truncated and point mutated versions of LytA result in loss of stability and enzymatic activity and folding [44,45]. Due to the similarity of the CBDs of pneumococcal phage lysins Pal and 23TH_48, we suspect that as shown for LytA, the catalytic domains alone are not sufficient for lytic activity and the CWBD is crucial for correct folding and lethality of the lysin.

Turbidity reduction assays and cell counts showed that the 23TH_48 lysin effectively reduced *S. pneumoniae* cell counts by up to 4 log_10_ within 1 h. These findings are comparable to previous results for turbidity reduction assays of the lysins Pal and Cpl-1, [18,20]. In studies with the pneumococcal lysins Pal and Cpl-1, different serotypes, as well as capsule-deficient strains, have been killed in the range from 3 to 5 log_10_ CFU/mL using 100 U/mL of lysin. Pal reduced the cell counts of an *S. pneumoniae* serotype 19F strain in 30 s by 4 log_10_ CFU/mL and the non-encapsulated R36A strain by 4.2 log_10_ CFU/mL using 100 U/mL [20]. The Cpl-1 lysin reduced the cell counts of an *S. pneumoniae* serotype 19F strain by 4.2 log_10_ CFU/mL and the R6 strain by 3.2 log_10_ CFU/mL using the same conditions [18]. Incubating the Cpl-1 lysin in a concentration of 5 ng/µL with the *S. pneumoniae* R6 strain for 60 min at 37 °C resulted in the eradication of the culture [46]. Testing the lysins Pal and LytA under the same conditions led to reductions of 5 and 7.5 log_10_ CFU/mL, respectively [39]. The chimeric lysin PL3 eliminated the R6 culture at a concentration of 0.5 ng/µL [47].

In this study, we showed that a high concentration of the 23TH_48 lysin is needed to prevent biofilms (228.8 ng/µL), while a relatively low concentration (14.3 ng/µL) could effectively disrupt preformed biofilms within 2 h compared to the untreated control. It is interesting to see the difference in the quantity of 23TH_48 required to prevent biofilm formation and that needed to disrupt a biofilm naive to 23TH_48. Previous work has investigated a mutant of the *S. pneumoniae* strain R6 used to examine the treatment of biofilms with other lysins similar to 23TH_48 [19]. In this study, *S. pneumoniae* P046 biofilms were treated for 4 h at 37 °C with 800 U/mL (160 U) lysin in 20 mM sodium phosphate buffer and the effect was evaluated with crystal violet staining. LytA reduced the biofilm by 80%, whereas Cpl-1 led to 50% reduction and Cpl-7 to a 70% reduction [19]. The Cpl-1 lysin was shown to reduce *S. pneumoniae* P046 biofilms (stained with crystal violet) by 50% in 2 h using a concentration of 1 ng/µL at 37 °C [46].

Phage lysin 23TH_48 could be a worthwhile candidate for further study as a potential therapeutic to treat pneumococcal infections. It may be possible to expand the lytic capabilities of 23TH_48 by using it in combination with other lysins, bacteriocins or antibiotics. Such combinations could improve the killing activity and broaden its bacterial spectrum of action. This approach has been shown to be very effective for other lysins targeting *S. pneumoniae*. Synergistic effects were reported for lysins Cpl-1 and Pal in vitro [48] and in a mouse model [49]. Furthermore, LytA and cefotaxime showed synergy in time-kill assays and fractional inhibitory concentrations (FICs) [50], while Cpl-1 and daptomycin were effective in a mouse model of pneumococcal bacteremia [51] Additionally, due to the modular structure of 23TH_48, protein engineering could be used to improve its host range, stability, and lytic activity. For instance, the exchange of catalytic domains, CWBDs and the linker modules between lysins could increase both lytic activity and antibacterial spectrum [52]. This approach has been tested successfully for the chimeric lysins PL3 [47], a construct consisting of the catalytic domain of Pal and the CBD of LytA [47]; and Cpl-711, comprising the catalytic domain of Cpl-7 and the CBD of Cpl-1 [46]. Both chimeric lysins were highly active against biofilms and in animal models and are currently the most active lysins against pneumococci compared to their parental enzymes [39,47,53]. Another successful approach to enhance killing activity is to engineer changes in the charge of the lysin, increasing its affinity to the bacterial cell surface [47].

## 4. Materials and Methods

### 4.1. Bacterial Strains

All strains used in this study (Table S7) were stored at −20 or −80 °C. *Streptococcus, Enterococcus*, *Bacillus,* and *Staphylococcus* strains were cultivated in BHI broth (Oxoid, Hampshire, UK). *Escherichia coli* for protein expression was grown in Luria-Bertani (LB) broth (Fisher Scientific, Waltham, MA, USA), supplemented with 50 µg/mL kanamycin (Sigma Aldrich, Saint Louis, MO, USA). Bacteria were incubated at 37 °C and shaken at 120 rpm, if necessary. *Lactococcus lactis* was cultivated in GM17 broth (Oxoid, Hampshire, UK) at 30 °C. The *Streptococcus infantis* phage 23TH and *Streptococcus anginosus* phage SA01 and their host strains *Streptococcus infantis* 23TH and *Streptococcus anginosus* SA01 were saliva isolates sourced from the APC Culture Collection (APC Microbiome Ireland, Cork, Ireland).

### 4.2. Plaque Assays and Phage Propagation

Plaque assays were carried out using BHI agar (1.5% *w*/*v*) and a BHI agar (0.4% *w/v*) overlay with calcium chloride (final concentration 10 mM). To determine phage titres, 10-fold serial dilutions of phage lysate (10^1^–10^7^) were prepared in SM buffer (50  mM Tris-HCl; 100  mM NaCl; 8.5  mM MgSO_4_; pH 7.5). Twenty µL of each dilution and 100 µL of an overnight culture of the respective host strain were added to 3 mL of molten BHI overlay agar and poured onto BHI agar plates. After incubation overnight at 37 °C, the phage titre was determined using plates with 30 to 250 plaques. To obtain high titre phage lysates, the plate lysis method was conducted as described previously [54]. Bacterial cultures were infected with phage lysates with different titres and poured on BHI plates in 3 mL molten top agarose (0.2% *w/v*). After incubation overnight, 3 mL SM buffer was added to the top layer and incubated for 24 h at room temperature (RT), shaking at 120 rpm. Top layers were removed and filtered through 0.45 µm filters after centrifugation for 10 min at 4500× *g*. Lysates were stored at 4 °C.

### 4.3. Efficiency of Lysogeny

The efficiency of lysogeny was determined by using a 10^10^ plaque forming unit per mL (PFU/mL) phage lysate of phage SA01 and a 10^9^ PFU/mL phage lysate of phage 23TH. To prepare phage seeded plates, 100 µL of phage lysate was spread evenly on BHI agar plates with glass beads and left to dry under a laminar hood for 10 min. Serial dilutions of an overnight culture of the respective host strain (10^1^–10^8^) were prepared. Afterward, 100 µL of dilutions 10^4^–10^8^ were added to 3 mL liquid BHI agar (0.2% *w/v*) overlay and plated onto phage seeded plates and plates not seeded with phage. After incubation at 37 °C for 24 h, CFUs were enumerated on the countable plates. The efficiency of lysogeny was calculated by dividing the number of CFUs on phage seeded plates by the number of CFUs on unseeded plates and multiplied by 100, resulting in per cent of lysogeny.

### 4.4. CsCl Purification of Phages for Transmission Electron Microscopy

Twenty milliliters of a high titre phage lysate (>1 × 10^9^ PFU/mL) were prepared for CsCl purification by adding to a final concentration 10 % *w/v* PEG-8000 (Sigma Aldrich, Saint Louis, MO, USA) and 0.5 M NaCl (Sigma Aldrich, Saint Louis, MO, USA). Samples were left at 4 °C overnight, followed by centrifugation at 4700× *g* for 20 min at 4 °C. After removing the supernatant, the remaining pellet was dried by inverting the tube for 5 min. The pellet was resuspended in 1 mL SM buffer and transferred to a new tube. An equal volume of chloroform (Sigma Aldrich, Saint Louis, MO, USA) was added, and the tube was vortexed for 30 s, followed by centrifugation at 2500× *g* for 5 min at RT. The aqueous phase was aspirated into a new tube, and the step of chloroform treatment was repeated until all the PEG residue was removed. The final phage preparation was concentrated by ultracentrifugation (34,000× *g* for 2.5 h at 4 °C) using a 3M CsCl (Sigma Aldrich, Saint Louis, MO, USA)and 5M CsCl gradient. The band containing the isolated phage was removed with a syringe and subjected to dialysis using deionized H_2_O and a Vivaspin 6, 10 KDa MWCO column (Sartorius, Göttingen, Germany). Purified whole phage was stored at 4 °C. A 10 µL aliquot of phage was placed on a Formvar/Carbon 200 mesh copper grid, Cu grids (Electron Microscopy Sciences, Hatfield, MA, USA) with subsequent removal of an excess sample by blotting. Grids were then negatively contrasted with 0.5% (*w/v*) uranyl acetate and examined at UCD Conway Imaging Core Facility (University College Dublin, Dublin, Ireland) by transmission electron microscope.

### 4.5. Viral DNA Extraction, Amplification, Library Preparation and Sequencing

A 20 mL phage lysate was mixed with 2 mL of 5 M NaCl and 2.2 g PEG-8000 (final concentration of 0.4 M NaCl and 10% (*w/v*) PEG) and stored at 4 °C on ice overnight. Samples were centrifuged at 4700*× g* for 20 min at 4 °C in a swing bucket rotor. Supernatants were removed, and pellets were dried for 5 min by inverting the tube. Pellets were resuspended in 400 μL SM buffer (50 mM Tris-HCl; 100 mM NaCl; 8.5 mM MgSO_4_; pH 7.5). An equal volume of chloroform was added and mixed for 30 s, followed by centrifugation at 2500× g for 5 min. The aqueous phase was aspirated into a new microcentrifuge tube. Forty microliters of 10 × Nuclease Buffer (50 mM CaCl_2_; 10 mM MgCl_2_), was added and the sample was incubated with 20 U of DNase I (Thermo Fisher, Waltham, MA, USA) and 10 U of RNase (Ambion, Carlsbad, CA, USA) for 1 h at 37 °C. Nucleases were inactivated at 70 °C for 10 min before samples were treated with 2 μL of freshly prepared 20 mg/μL Proteinase K (Sigma Aldrich, Saint Louis, MO, USA) and 20 μL 10% sodium dodecyl sulfate (SDS) (Sigma Aldrich, Saint Louis, MO, USA) for 20 min at 56 °C. One hundred microliters of phage lysis buffer were added, and the mix was incubated at 65 °C for 10 min. An equal volume of phenol:chloroform: isoamyl alcohol (25:24:1) (Sigma Aldrich, Saint Louis, MO, USA) was added and centrifuged at 8000 g for 5 min. This step was repeated, and the aqueous phase was further purified with the Quiagen Blood and Tissue purification kit (Quiagen, Hilden, Germany) following the manufacturer’s instructions. Viral DNA was recovered by passing elution buffer (50 µL) twice through a column to maximize yield. Both viral genomes were amplified using Genomiphi V2 KIT (GE Healthcare, Chicago, IL, USA). Library preparation of 23TH was performed using the TruSeq DNA Nano Library Prep Nano Kit (Illumina, San Diego, CA, USA) according to the manufacturer’s instructions, while the genome of SA01 was prepared for sequencing using the Nextera XT DNA Library Kit (Illumina, San Diego, CA, USA). Library quality was assessed using the Agilent Bioanalyzer (Agilent Technologies, Santa Clara, CA, USA) and via Qubit^®^ 3.0 Fluorometer (Thermo Fisher, Waltham, MA, USA) measurements. Genomic sequencing was performed on an Illumina HiSeq 2500 platform at GATC Biotech AG, Germany. Reads were assembled using metaSPAdes [55].

### 4.6. Bioinformatic Analysis of Genomes

ORFs of 23TH and SA01 were predicted with RASTtk (https://rast.nmpdr.org/rast.cgi, [56]). Functional inferences for predicted ORF gene products were obtained by searches conducted using BLASTP (http://blast.ncbi.nlm.nih.gov/Blast.cgi?PAGE=Proteins), Pfam (http://pfam.xfam.org/search#tabview=tab1; [57]), InterProScan (https://www.ncbi.nlm.nih.gov/pmc/articles/PMC3998142/; [58]) and HHpred (https://toolkit.tuebingen.mpg.de/#/tools/hhpred; [59]). Additionally, translated ORFs from phages were searched against hidden Markov model profiles downloaded from the Prokaryotic Virus Orthologous Groups (pVOGs) database [60] using hmmscan (v.3.1b2) [61] with an E-value cut off of 1 × 10 ^−5^. Transmembrane domains and lipoprotein cleavage signals were identified using TMHMM v.2 (http://www.cbs.dtu.dk/services/TMHMM/; [62]) and LipoP v.1 (http://www.cbs.dtu.dk/services/LipoP/; [63]), respectively. The molecular weight of the predicted ORFs was estimated using the batch protein molecular weight determination of the sequence manipulation suite (http://www.bioinformatics.org/sms2/protein_mw.html). The presence of transfer RNA genes was investigated with the use of tRNAscan-SE (http://lowelab.ucsc.edu/tRNAscan-SE/; [64]) and ARAGORN (http://130.235.46.10/ARAGORN/; [65]).

### 4.7. Cloning of Lysin Genes

The lysin genes were amplified from phage gDNA by PCR amplification using primers SA01_53FBamHI and SA01_53RHindIII for SA01_53, and primers 23THFBamHI and 23THRHindIII for 23TH_48 (Table S8). The PCR products were cloned into the vector pET-28b(+) using restriction digests with BamHI and HindII (New England Biolabs, Ipswich, MA, USA) and ligation with T4 ligase (New England Biolabs, Ipswich, MA, USA) to obtain the constructs pET-28b(+)SA01_53 and pET-28b(+)23TH_48 in which the His-tag was located at the 5′ end of the lysin gene (Table S9). The plasmids were transformed into One Shot TOP10 chemically competent cells (Life Technologies, Carlsbad, CA, USA) and propagated on LB plates supplemented with kanamycin (50 µg/mL). All constructs were validated by DNA sequencing using universal T7 primers (Table S8). After confirmation of sequence integrity, plasmids were transformed into One Shot BL21 (DE3) chemically competent cells (Life Technologies, Carlsbad, CA, USA) for subsequent protein expression.

### 4.8. Expression and Purification

Cells were grown in LB media containing 50 µg/mL kanamycin to an OD_600_ of 0.6. After induction of expression with isopropyl-β-D-thiogalactopyranoside (IPTG) (Sigma Aldrich, Saint Louis, MO, USA) at a final concentration of 10 mM, cells were left overnight at RT. Cells were centrifuged and resuspended in Binding Buffer (50 mM sodium phosphate pH 7.4, 300 mM sodium chloride, 10 mM imidazole). For small culture volumes (25 mL), cells were disrupted using FastPrep (MP Biomedicals, Solon, OH, USA) followed by centrifugation and purification with His-Spin Protein Miniprep^™^ Columns (Zymo Research, Irvine, CA, USA).

For larger culture volumes, cells were sonicated on ice, and the supernatant was collected by centrifugation using sequential spins at 10,000× g for 30 min at 4 °C. Purification was done with a His GraviTrap (GE Healthcare, Chicago, IL, USA) column using a Washing Buffer (50 mM sodium phosphate pH 7.4, 300 mM sodium chloride, 50 mM imidazole) and Elution Buffer (50 mM sodium phosphate pH 7.4, 300 mM sodium chloride, 250 mM imidazole). All fractions were analyzed on Bolt 4–12% Bis-Tris gels (Invitrogen, Carlsbad, CA, USA). Elution fractions were pooled, filtered (0.45 µm) and stored at 4 °C. Imidazole was removed by using 5 mL Zeba™ Spin Desalting Columns (7K MWCO, Thermo Fisher, Waltham, MA, USA) and protein concentrations were quantified with a NanoDrop Microvolume Spectrophotometer (Thermo Fisher, Waltham, MA, USA). Purified proteins were stored at 4 °C until required.

### 4.9. Screening for Lytic Activity and Host Range of Lysins

To determine the host range of lysins, 100 µL of a test culture was added to 3 mL of its growth media containing 0.2% *w/v* agarose and overlaid an agar plate (1.5% *w/v*) of the same media. Ten µL of the expressed lysins were then spotted on the agarose layer once set. Plates were then incubated at 37 °C overnight and observed for lysis the following day.

### 4.10. In Vitro Quantification of Lysin 23TH_48 Activity Against S. pneumoniae

The lytic activity of lysin 23TH_48 was tested using turbidity reduction assays. Overnight cultures of *S. pneumoniae* cells were sub-cultured (2% inoculum) and grown to an OD_600_ of ~0.6. Subsequently, the cells were harvested by centrifugation (5000× *g*, 5 min) before washing twice with PBS and final resuspension in SPB pH 7.4 to reach an OD_600_ 0.6–0.8. The activity was measured by mixing 180 µL cell suspension with 20 µL of 23TH_48 in a 96 well plate (Sarstedt, Newton NC, USA) and changes in optical density (OD_595_) were monitored immediately in subsequent minutes using a microplate reader (Thermo Fisher, Waltham, MA, USA) at 37 °C for 1h. Buffer alone was added to cells in the control wells.

To determine one activity unit (1U), serial dilutions of 23TH_48 were prepared in SPB pH 7.4. For this manuscript, 1U is the lysin concentration that decreases optical density by half in 15 min, as previously described [18].

Bacterial viability was determined by preparing *S. pneumoniae* cells as described above and resuspending the cells in SPB pH 7.4 to an OD_600_ of 0.6–0.8 Cells were subsequently exposed to 23TH_48 (final concentration *S. pneumoniae* DSM 24048: 1.5 ng/µL, *S. pneumoniae* R6: 120 ng/µL). For cell counts, 20 µL aliquots were taken at the following time points (15, 30, and 60 min) and serially diluted in PBS. Ten µL of these dilutions were spotted on BHI agar plates and incubated overnight at 37 °C, CFU counts were then taken. All experiments were performed in triplicate.

### 4.11. Biofilm Assays S. pneumoniae R6

For biofilm assays, overnight cultures of *S. pneumoniae* R6 were inoculated into fresh BHI media until cells reached mid-log phase. Cells were spun down and resuspended in fresh BHI media to reach an OD_600_ of 0.5 and diluted by a further 1/100. For biofilm formation, 200 µL of diluted bacterial culture was added to the wells of Costar 3595 96-well PST microtiter plates (Corning, Corning, NY, USA) and incubated at 34 °C overnight. The next day, the liquid was taken out of wells and biofilms were washed once with 200 µL PBS. The 23TH_48 lysin was added in different test concentrations to 180 µL fresh BHI media in the wells to a final volume of 200 µL and incubated for 2 h at 37 °C. Additionally, the capability of preventing *S. pneumoniae* R6 biofilm formation was tested by adding the 23TH_48 before incubation of cells overnight. Therefore, 20 µL of different lysin concentrations were added to 180 µL of the diluted cultures as described above, followed by incubation at 34 °C overnight. For quantification of biofilms, an XTT/menadione assay was carried out. This assay relies on the reduction of XTT by metabolically active cells to an orange/yellow water-soluble formazan derivative that can be quantified colorimetrically and correlate to cell viability [66]. The XTT/menadione solution was prepared by adding 0.01 g XTT (Abcam, Cambridge, UK) to 20 mL of water followed by filter sterilization using a 0.22 µm filter. Ten microliters of a 10 mM menadione (Sigma Aldrich, Saint Louis, MO, USA) acetone solution was added to the XTT solution and frozen at −80 °C until further use. Just before XTT/menadione assays, the solution was thawed, and 100 µL were added to each well of the 96-well plate after washing once with 200 µL PBS. The 96 well plates were incubated in the dark at 37 °C for 2 h. Afterwards, the absorbance was measured at a wavelength of 492 nm in a plate reader. Experiments were carried out with six replicates for each condition.

### 4.12. Statistical Analysis

Statistical analysis to determine differences between means was done with GraphPadPrism 8.0.1. Cell counts of the time-kill curves were analyzed using the multiple t-test, whereas for biofilm assays one-way ANOVA and multiple comparisons were applied with a Dunnet’s post-test.

### 4.13. Accession Number

The genome sequence of *Streptococcus* phages 23TH and SA01 were submitted to GenBank under accession numbers MT900487 and MT900488, respectively.

## 5. Conclusions

In conclusion, we identified two new Streptococcal phages from the oral microbiome, 23TH and SA01. Their lysins, 23TH_48 and SA01_53, were recombinantly expressed, characterized and tested for their lethality. SA01_53 was found to only lyse its host strain of *S. anginosus*, while 23TH_48 was found to possess a broader lytic activity beyond its host strain of *S. infantis*, with several *S. pneumoniae* isolates highly sensitive to its lytic activity. Given this activity, 23TH_48 could prove to be a promising candidate to help combat pneumococcal infections.

## Figures and Tables

**Table 1:** Host range of lysin 23TH_48 tested by spotting on bacterial lawns.

Genus	Species	Tested Isolates	23TH_48	
*Streptococcus*	*agalactiae*	2	-	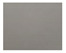
*Streptococcus*	*lutentiensis*	10	-	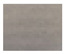
*Streptococcus*	*infantis*	1	+	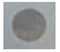
*Streptococcus*	*anginosus*	1	-	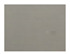
*Streptococcus*	*pneumoniae*	6	+	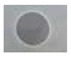
*Streptococcus*	*sanguinis*	1	-	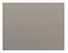
*Streptococcus*	*hyointestinalis*	1	-	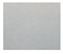
*Streptococcus*	*pyogenes*	2	-	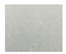
*Streptococcus*	*dysgalactiae*	4	-	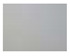
*Streptococcus*	*bovis*	3	-	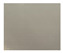
*Streptococcus*	*mutans*	2	*-*	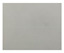
*Streptococcus*	*uberis*	6	*-*	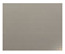
*Streptococcus*	*infantarius*	1	*-*	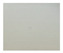
*Streptococcus*	*salivarius*	5	*-*	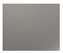
*Lactococcus*	*lactis*	1	*-*	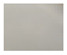
*Staphylococcus*	*aureus*	1	*-*	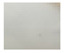
*Enterococcus*	*faecalis*	1	*-*	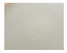
*Bacillus*	*cereus*	1	-	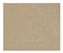

## Supplementary Materials

The following are available online at https://www.mdpi.com/1424-8247/13/12/478/s1, Figure S1: (A) Comparison of phage SA01 with its top five BLASTN hits. (B) Gegenees TBLASTX heat map analysis of the phages. Figure S2: (A) Comparison of phage 23TH with its top five BLASTN hits. (B) Gegenees TBLASTX heat map analysis of the phages. Figure S3: Phylogenetic trees of BlastP hits for lysin SA01_53 and 23TH_48. Figure S4: Alignment of amino acid sequences of 23TH_48 lysin with Pal lysin of *Streptococcus* phage Dp-1. Figure S5: Alignment of amino acid sequences of SA01_53 lysin with Cpl-7 lysin of *Streptococcus* phage Cp-7. Figure S6: SDS-PAGE of His-tag affinity chromatography purification of SA01_53. Figure S7: SDS-PAGE of His-tag affinity chromatography purification of recombinant 23TH_48. Figure S8: Time kill curves and respective cell counts for treatment of *S. pneumoniae* DSM 24048 and *S. pneumoniae* R6 cells with lysin 23TH_48. Table S1: Genbank details of closest phage related to *Streptococcus* phage SA01. Table S2: Details of *Streptococcus* phages found to possess homology at the protein level with *Streptococcus* phage SA01. Table S3: Genbank details of closest phages related to *Streptococcus* phage 23TH. Table S4: Details of *Streptococcus* phages found to possess homology at the protein level with *Streptococcus* phage 23TH. Table S5. Annotation of Streptococcus phage 23TH. Table S6. Annotation of Streptococcus phage SA01. Table S7: Bacterial strains used in the study. Table S8: Primers used in the study. Table S9: Plasmids constructed in the study.

## References

[1] Abranches J., Zeng L., Kajfasz J.K., Palmer S.R., Chakraborty B., Wen Z.T., Richards V.P., Brady L.J., Lemos J.A. (2018). Biology of Oral Streptococci. Microbiol. Spectr..

[2] Walker C.L.F., Rudan I., Liu L., Nair H., Theodoratou E., Bhutta Z.A., O’Brien K.L., Campbell H., Black R.E. (2013). Global Burden of Childhood Pneumonia and Diarrhoea. Lancet.

[3] World Health Organization Top 10 Causes of Death. https://www.who.int/gho/mortality_burden_disease/causes_death/top_10/en/.

[4] Rodrigues C.M.C., Groves H. (2017). Community-Acquired Pneumonia in Children: The Challenges of Microbiological Diagnosis. J. Clin. Microbiol..

[5] Geno K.A., Gilbert G.L., Song J.Y., Skovsted I.C., Klugman K.P., Jones C., Konradsen H.B., Nahm M.H. (2015). Pneumococcal Capsules and Their Types: Past, Present, and Future. Clin. Microbiol. Rev..

[6] Klugman K.P. (2009). The Significance of Serotype Replacement for Pneumococcal Disease and Antibiotic Resistance. Hot Topics in Infection and Immunity in Children V.

[7] Keller L.E., Robinson D.A., McDaniel L.S. (2016). Nonencapsulated Streptococcus Pneumoniae: Emergence and Pathogenesis. MBio.

[8] Cherazard R., Epstein M., Doan T.-L., Salim T., Bharti S., Smith M.A. (2017). Antimicrobial Resistant Streptococcus Pneumoniae. Am. J. Ther..

[9] Fischetti V. (2018). Development of Phage Lysins as Novel Therapeutics: A Historical Perspective. Viruses.

[10] O’Flaherty S., Coffey A., Meaney W., Fitzgerald G.F., Ross R.P. (2005). The Recombinant Phage Lysin LysK Has a Broad Spectrum of Lytic Activity against Clinically Relevant Staphylococci, Including Methicillin-Resistant Staphylococcus Aureus. J. Bacteriol..

[11] Nelson D., Schuch R., Chahales P., Zhu S., Fischetti V.A. (2006). PlyC: A Multimeric Bacteriophage Lysin. Proc. Natl. Acad. Sci. USA.

[12] Oechslin F., Daraspe J., Giddey M., Moreillon P., Resch G. (2013). In Vitro Characterization of PlySK1249, a Novel Phage Lysin, and Assessment of Its Antibacterial Activity in a Mouse Model of Streptococcus Agalactiae Bacteremia. Antimicrob. Agents Chemother..

[13] Briers Y., Volckaert G., Cornelissen A., Lagaert S., Michiels C.W., Hertveldt K., Lavigne R. (2007). Muralytic Activity and Modular Structure of the Endolysins of Pseudomonas Aeruginosa Bacteriophages? KZ and EL. Mol. Microbiol..

[14] Lood R., Winer B.Y., Pelzek A.J., Diez-Martinez R., Thandar M., Euler C.W., Schuch R., Fischetti V.A. (2015). Novel Phage Lysin Capable of Killing the Multidrug-Resistant Gram-Negative Bacterium Acinetobacter Baumannii in a Mouse Bacteremia Model. Antimicrob. Agents Chemother..

[15] Vázquez R., García P. (2019). Synergy Between Two Chimeric Lysins to Kill Streptococcus Pneumoniae. Front. Microbiol..

[16] Bustamante N., Campillo N.E., García E., Gallego C., Pera B., Diakun G.P., Sáiz J.L., García P., Fernando Díaz J., Menéndez M. (2010). Cpl-7, a Lysozyme Encoded by a Pneumococcal Bacteriophage with a Novel Cell Wall-Binding Motif. J. Biol. Chem..

[17] Bustamante N., Iglesias-Bexiga M., Bernardo-García N., Silva-Martín N., García G., Campanero-Rhodes M.A., García E., Usón I., Buey R.M., García P. (2017). Deciphering How Cpl-7 Cell Wall-Binding Repeats Recognize the Bacterial Peptidoglycan. Sci. Rep..

[18] Loeffler J.M., Djurkovic S., Fischetti V.A. (2003). Phage Lytic Enzyme Cpl-1 as a Novel Antimicrobial for Pneumococcal Bacteremia. Infect. Immun..

[19] Domenech M., García E., Moscoso M., Garciá E., Moscoso M. (2011). In Vitro Destruction of Streptococcus Pneumoniae Biofilms with Bacterial and Phage Peptidoglycan Hydrolases. Antimicrob. Agents Chemother..

[20] Loeffler J.M., Nelson D., Fischetti V.A. (2001). Rapid Killing of Streptococcus Pneumoniae with a Bacteriophage Cell Wall Hydrolase. Science.

[21] Vázquez R., García E., García P. (2018). Phage Lysins for Fighting Bacterial Respiratory Infections: A New Generation of Antimicrobials. Front. Immunol..

[22] NCBI Genome Information S. anginosus.

[23] Brueggemann A.B., Harrold C.L., Rezaei Javan R., Van Tonder A.J., McDonnell A.J., Edwards B.A. (2017). Pneumococcal Prophages Are Diverse, but Not without Structure or History. Sci. Rep..

[24] NCBI Genome Information S. infantis.

[25] Dalmasso M., de Haas E., Neve H., Strain R., Cousin F.J., Stockdale S.R., Ross R.P., Hill C. (2015). Isolation of a Novel Phage with Activity against Streptococcus Mutans Biofilms. PLoS ONE.

[26] Cheng Q., Fischetti V.A. (2007). Mutagenesis of a Bacteriophage Lytic Enzyme PlyGBS Significantly Increases Its Antibacterial Activity against Group B Streptococci. Appl. Microbiol. Biotechnol..

[27] Gaeng S., Scherer S., Neve H., Loessner M.J. (2000). Gene Cloning and Expression and Secretion of Listeria Monocytogenes Bacteriophage-Lytic Enzymes in Lactococcus Lactis. Appl. Environ. Microbiol..

[28] Low L.Y., Yang C., Perego M., Osterman A., Liddington R.C. (2005). Structure and Lytic Activity of a Bacillus Anthracis Prophage Endolysin. J. Biol. Chem..

[29] Sass P., Bierbaum G. (2007). Lytic Activity of Recombinant Bacteriophage Φ11 and Φ12 Endolysins on Whole Cells and Biofilms of Staphylococcus aureus. Appl. Environ. Microbiol..

[30] Loessner M.J., Kramer K., Ebel F., Scherer S. (2002). C-Terminal Domains of Listeria Monocytogenes Bacteriophage Murein Hydrolases Determine Specific Recognition and High-Affinity Binding to Bacterial Cell Wall Carbohydrates. Mol. Microbiol..

[31] Zimmer M., Vukov N., Scherer S., Loessner M.J. (2002). The Murein Hydrolase of the Bacteriophage Φ3626 Dual Lysis System Is Active against All Tested Clostridium Perfringens Strains. Appl. Environ. Microbiol..

[32] Maestro B., Sanz J.M. (2016). Choline Binding Proteins from Streptococcus Pneumoniae: A Dual Role as Enzybiotics and Targets for the Design of New Antimicrobials. Antibiotics.

[33] Hall-Stoodley L., Hu F.Z., Gieseke A., Nistico L., Nguyen D., Hayes J., Forbes M., Greenberg D.P., Dice B., Burrows A. (2006). Direct Detection of Bacterial Biofilms on the Middle-Ear Mucosa of Children with Chronic Otitis Media. J. Am. Med. Assoc..

[34] Coates H., Thornton R., Langlands J., Filion P., Keil A.D., Vijayasekaran S., Richmond P. (2008). The Role of Chronic Infection in Children with Otitis Media with Effusion: Evidence for Intracellular Persistence of Bacteria. Otolaryngol. Head Neck Surg..

[35] Sanderson A.R., Leid J.G., Hunsaker D. (2006). Bacterial Biofilms on the Sinus Mucosa of Human Subjects with Chronic Rhinosinusitis. Laryngoscope.

[36] Domenech M., García E., Moscoso M. (2012). Biofilm Formation in Streptococcus Pneumoniae. Microbial Biotechnol..

[37] McCullers J.A., Karlström Å., Iverson A.R., Loeffler J.M., Fischetti V.A. (2007). Novel Strategy to Prevent Otitis Media Caused by Colonizing Streptococcus Pneumoniae. PLoS Pathog..

[38] Walker P.J., Siddell S.G., Lefkowitz E.J., Mushegian A.R., Dempsey D.M., Dutilh B.E., Harrach B., Harrison R.L., Hendrickson R.C., Junglen S. (2019). Changes to Virus Taxonomy and the International Code of Virus Classification and Nomenclature Ratified by the International Committee on Taxonomy of Viruses (2019). Arch. Virol..

[39] Díez-Martínez R., De Paz H., Bustamante N., García E., Menéndez M., García P. (2013). Improving the Lethal Effect of Cpl-7, a Pneumococcal Phage Lysozyme with Broad Bactericidal Activity, by Inverting the Net Charge of Its Cell Wall-Binding Module. Antimicrob. Agents Chemother..

[40] Pohane A.A., Patidar N.D., Jain V. (2015). Modulation of Domain-Domain Interaction and Protein Function by a Charged Linker: A Case Study of Mycobacteriophage D29 Endolysin. FEBS Lett..

[41] Schmelcher M., Tchang V.S., Loessner M.J. (2011). Domain Shuffling and Module Engineering of *Listeria* Phage Endolysins for Enhanced Lytic Activity and Binding Affinity. Microb. Biotechnol..

[42] Gräslund S., Nordlund P., Weigelt J., Hallberg B.M., Bray J., Gileadi O., Knapp S., Oppermann U., Arrowsmith C., Hui R. (2008). Protein Production and Purification. Nat. Methods.

[43] Pimenta F., Gertz R.E., Park S.H., Kim E., Moura I., Milucky J., Rouphael N., Farley M.M., Harrison L.H., Bennett N.M. (2019). Streptococcus infantis, Streptococcus Mitis, and Streptococcus Oralis Strains with Highly Similar Cps5 Loci and Antigenic Relatedness to Serotype 5 Pneumococci. Front. Microbiol..

[44] Garcia J.L., Diaz E., Romero A., Garcia P. (1994). Carboxy-Terminal Deletion Analysis of the Major Pneumococcal Autolysin. J. Bacteriol..

[45] Romero P., López R., García E. (2007). Key Role of Amino Acid Residues in the Dimerization and Catalytic Activation of the Autolysin LytA, an Important Virulence Factor in Streptococcus Pneumoniae. J. Biol. Chem..

[46] Díez-Martínez R., De Paz H.D., García-Fernández E., Bustamante N., Euler C.W., Fischetti V.A., Menendez M., García P. (2014). A Novel Chimeric Phage Lysin with High in Vitro and in Vivo Bactericidal Activity against Streptococcus Pneumoniae. J. Antimicrob. Chemother..

[47] Blázquez B., Fresco-Taboada A., Iglesias-Bexiga M., Menéndez M., García P. (2016). PL3 Amidase, a Tailor-Made Lysin Constructed by Domain Shuffling with Potent Killing Activity against Pneumococci and Related Species. Front. Microbiol..

[48] Loeffler J.M., Fischetti V.A. (2003). Synergistic Lethal Effect of a Combination of Phage Lytic Enzymes with Different Activities on Penicillin-Sensitive and -Resistant Streptococcus Pneumoniae Strains. Antimicrob. Agents Chemother..

[49] Jado I. (2003). Phage Lytic Enzymes as Therapy for Antibiotic-Resistant Streptococcus Pneumoniae Infection in a Murine Sepsis Model. J. Antimicrob. Chemother..

[50] Rodríguez-Cerrato V., García P., del Prado G., García E., Gracia M., Huelves L., Ponte C., López R., Soriano F. (2007). In Vitro Interactions of LytA, the Major Pneumococcal Autolysin, with Two Bacteriophage Lytic Enzymes (Cpl-1 and Pal), Cefotaxime and Moxifloxacin against Antibiotic-Susceptible and -Resistant Streptococcus Pneumoniae Strains. J. Antimicrob. Chemother..

[51] Vouillamoz J., Entenza J.M., Giddey M., Fischetti V.A., Moreillon P., Resch G. (2013). Bactericidal Synergism between Daptomycin and the Phage Lysin Cpl-1 in a Mouse Model of Pneumococcal Bacteraemia. Int. J. Antimicrob. Agents.

[52] Diaz E., Lopez R., Garcia J.L. (1991). Chimeric Pneumococcal Cell Wall Lytic Enzymes Reveal Important Physiological and Evolutionary Traits. J. Biol. Chem..

[53] Corsini B., Díez-Martínez R., Aguinagalde L., González-Camacho F., García-Fernández E., Letrado P., García P., Yuste J. (2018). Chemotherapy with Phage Lysins Reduces Pneumococcal Colonization of the Respiratory Tract. Antimicrob. Agents Chemother..

[54] Sambrook J., Fritsch E.F., Maniatis T. (1989). Molecular Cloning: A Laboratory Manual.

[55] Nurk S., Meleshko D., Korobeynikov A., Pevzner P.A. (2017). MetaSPAdes: A New Versatile Metagenomic Assembler. Genome Res..

[56] Brettin T., Davis J.J., Disz T., Edwards R.A., Gerdes S., Olsen G.J., Olson R., Overbeek R., Parrello B., Pusch G.D. (2015). RASTtk: A Modular and Extensible Implementation of the RAST Algorithm for Building Custom Annotation Pipelines and Annotating Batches of Genomes. Sci. Rep..

[57] Finn R.D., Coggill P., Eberhardt R.Y., Eddy S.R., Mistry J., Mitchell A.L., Potter S.C., Punta M., Qureshi M., Sangrador-Vegas A. (2016). The Pfam Protein Families Database: Towards a More Sustainable Future. Nucleic Acids Res..

[58] Mitchell A., Chang H.-Y., Daugherty L., Fraser M., Hunter S., Lopez R., McAnulla C., McMenamin C., Nuka G., Pesseat S. (2015). The InterPro Protein Families Database: The Classification Resource after 15 Years. Nucleic Acids Res..

[59] Soding J., Biegert A., Lupas A.N. (2005). The HHpred Interactive Server for Protein Homology Detection and Structure Prediction. Nucleic Acids Res..

[60] Grazziotin A.L., Koonin E.V., Kristensen D.M. (2017). Prokaryotic Virus Orthologous Groups (PVOGs): A Resource for Comparative Genomics and Protein Family Annotation. Nucleic Acids Res..

[61] Eddy S.R. (2011). Accelerated Profile HMM Searches. PLoS Comput. Biol..

[62] Krogh A., Larsson B., von Heijne G., Sonnhammer E.L. (2001). Predicting Transmembrane Protein Topology with a Hidden Markov Model: Application to Complete Genomes. J. Mol. Biol..

[63] Juncker A.S., Willenbrock H., von Heijne G., Brunak S., Nielsen H., Krogh A. (2003). Prediction of Lipoprotein Signal Peptides in Gram-Negative Bacteria. Protein Sci..

[64] Lowe T.M., Eddy S.R. (1997). TRNAscan-SE: A Program for Improved Detection of Transfer RNA Genes in Genomic Sequence. Nucleic Acids Res..

[65] Laslett D. (2004). ARAGORN, a Program to Detect TRNA Genes and TmRNA Genes in Nucleotide Sequences. Nucleic Acids Res..

[66] Tunney M.M., Ramage G., Field T.R., Moriarty T.F., Storey D.G. (2004). Rapid Colorimetric Assay for Antimicrobial Susceptibility Testing of Pseudomonas aeruginosa. Antimicrob. Agents Chemother..

