# Supplementary material for: A New Phage Lysin Isolated from the Oral Microbiome Targeting Streptococcus pneumoniae
Source: Pharmaceuticals (Basel). 2020 Dec 19;13(12):478. doi: 10.3390/ph13120478 (PMC7767030; doi:10.3390/ph13120478)
Supplement: Supplementary file 1 [file pharmaceuticals-13-00478-s001.zip › Supplements.docx]

**Supplements**


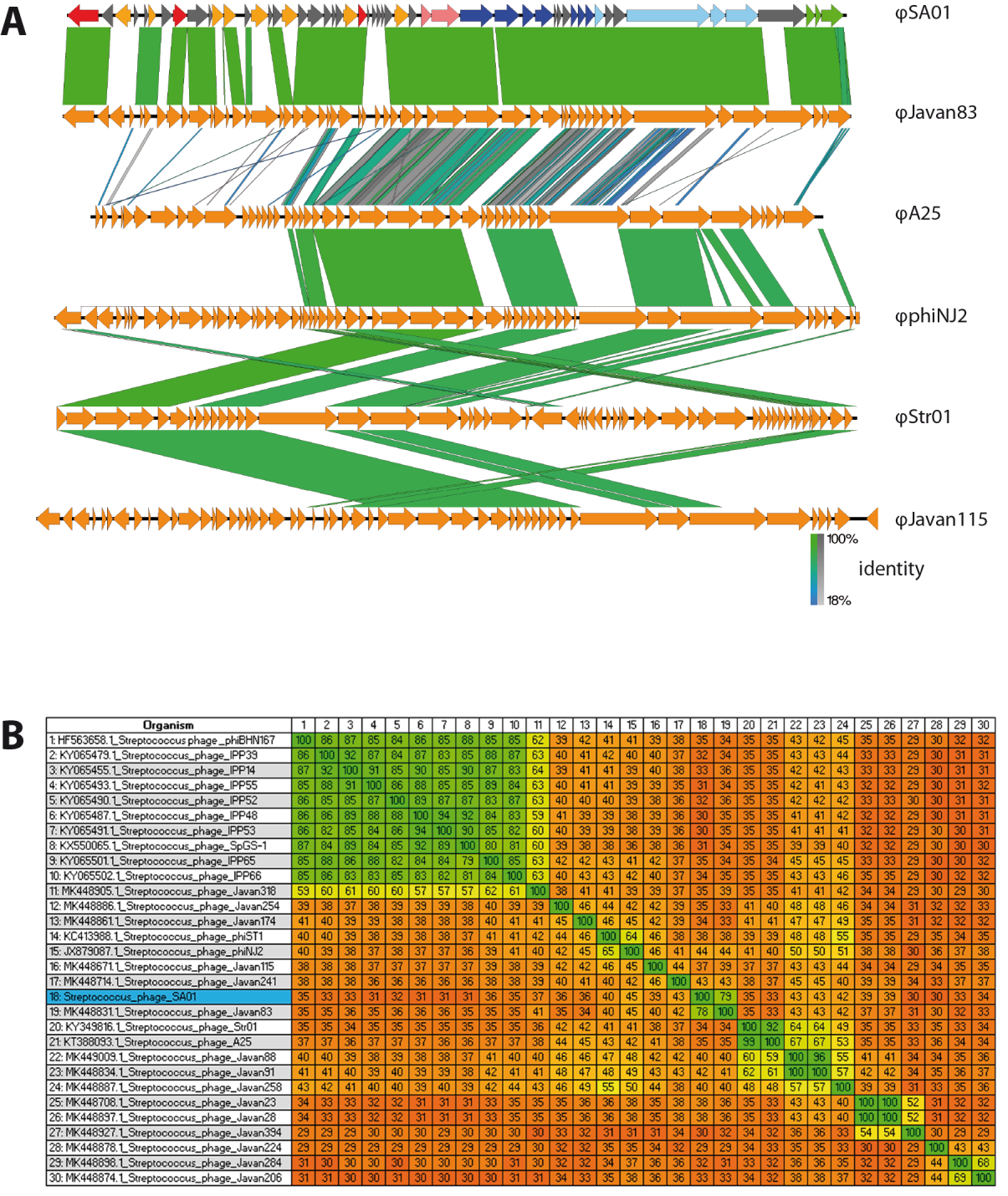


**Figure S1.** (**A**) Comparison of phage SA01 with its top five BLASTN hits and visualized with Easyfig. Genome maps comprise of arrows indicating the locations of open reading frames (ORFs) among different phage genomes. Lines between genome maps indicate levels of homology. Arrows for phage SA01 have been color-coded describing their predicted role: hypothetical proteins (grey), DNA replication and regulation proteins (orange), proteins involved in recombination (red), packaging proteins (light red), phage capsid proteins (dark blue), phage tail proteins (light blue) and lysis proteins (green). (**B**) Gegenees TBLASTX heat map analysis of the phages, with homology to the *Streptococcus* phage SA01 (highlighted in blue). Using accurate parameters, fragment length: 200 bp; and step size: 100 bp with the threshold set to 0%.


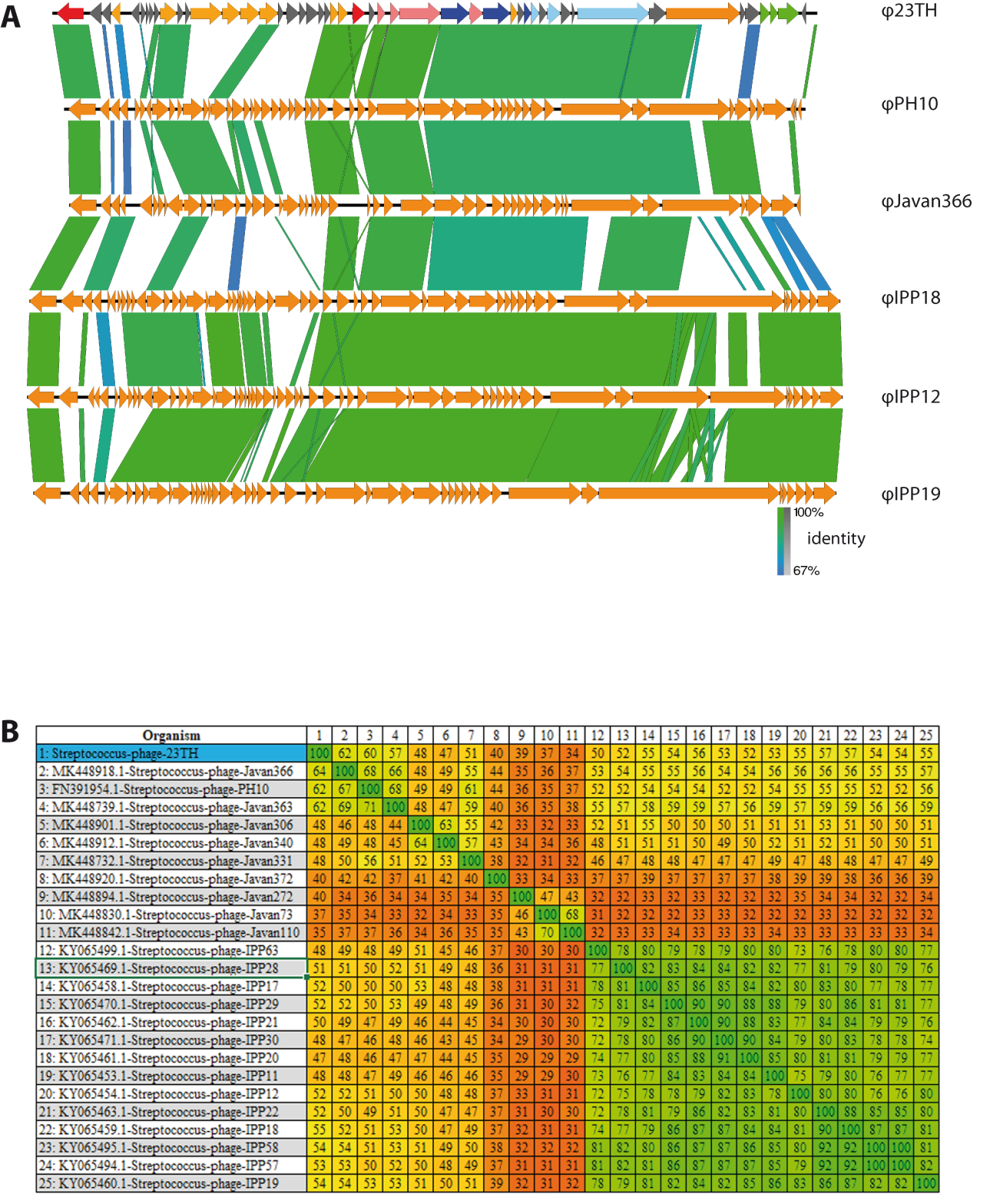


**Figure S2.** (**A**) Comparison of phage 23TH with its top five BLASTN hits and visualized with Easyfig. Genome maps comprise of arrows indicating the locations of open reading frames (ORFs) among different phage genomes. Lines between genome maps indicate levels of homology. Arrows for phage 23TH have been color-coded describing their predicted role: hypothetical proteins (grey), DNA replication and regulation proteins (orange), proteins involved in recombination (red), packaging proteins (light red), phage capsid proteins (dark blue), phage tail proteins (light blue) and lysis proteins (green). (**B**) Gegenees TBLASTX heat map analysis of the phages, with homology to the *Streptococcus* phage 23TH (highlighted in blue). Using accurate parameters, fragment length: 200 bp; and step size: 100 bp with the threshold set to 0%.


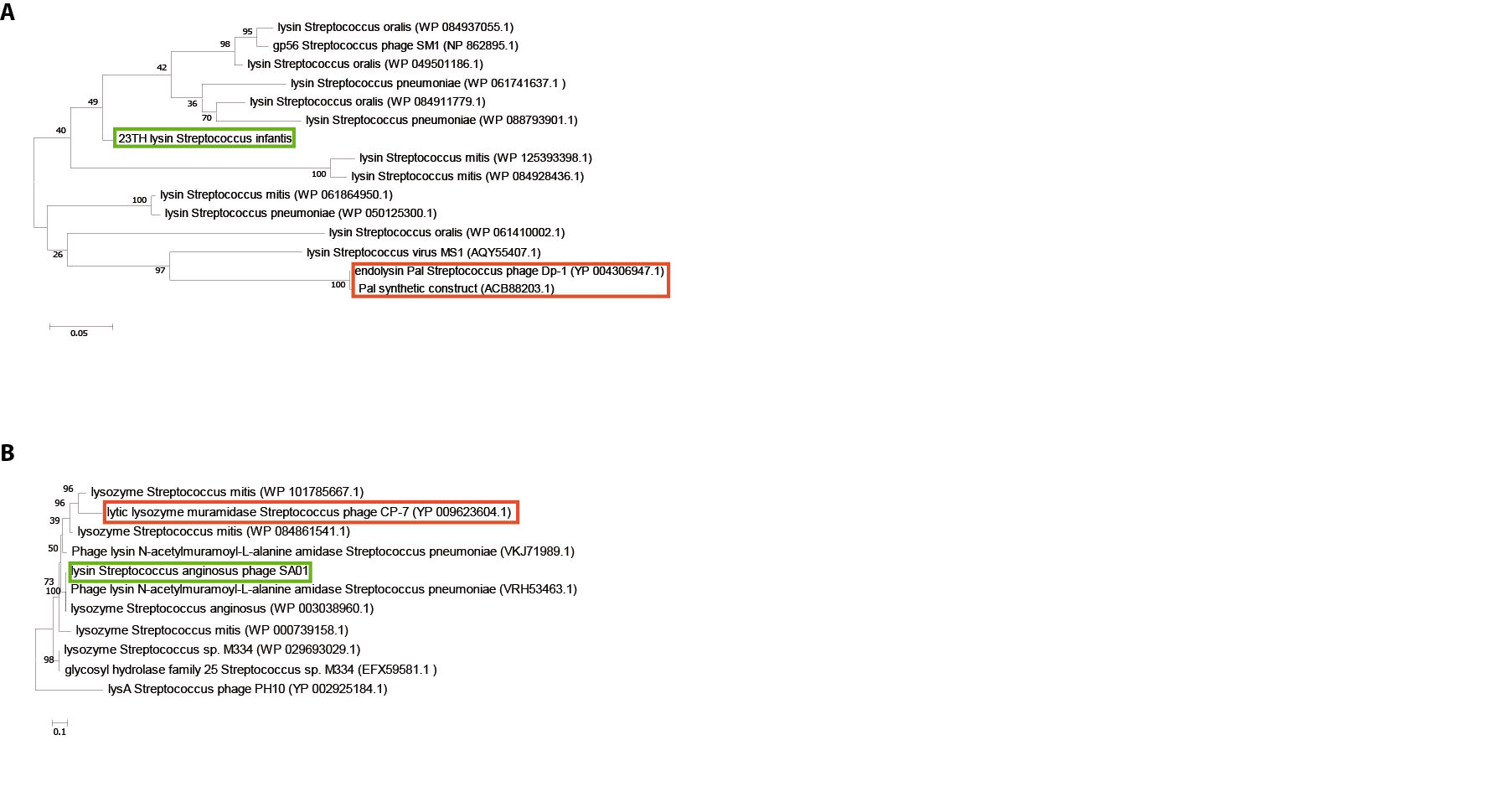


**Figure S3.** Phylogenetic trees (Neighbour-Joining Method) created with MEGA-X and number of bootstraps: 1000. (**A**) Top BlastP hits for 23TH_48 lysin. (**B**) Top BlastP hits for SA01_53 lysin.


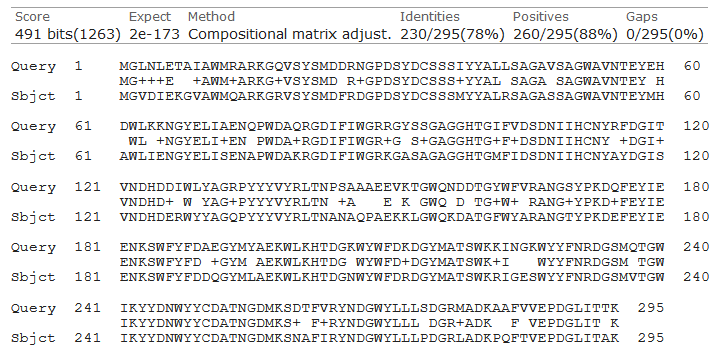


**Figure S4**. Alignment of amino acid sequences of 23TH_48 lysin (Query) with Pal lysin of *Streptococcus* phage Dp-1 (Sbjct). Use of Compositional matrix adjustment from NCBI BlastP.


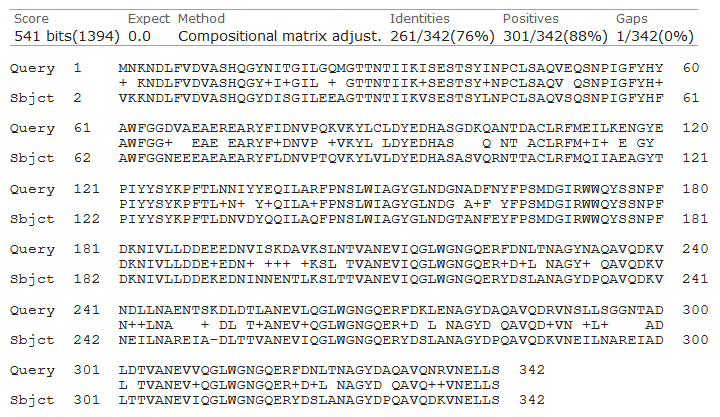


**Figure S5.** Alignment of amino acid sequences of SA01_53 lysin (Query) with Cpl-7 lysin of *Streptococcus* phage Cp-7 (Sbjct). Use of Compositional matrix adjustment from NCBI BlastP.

**
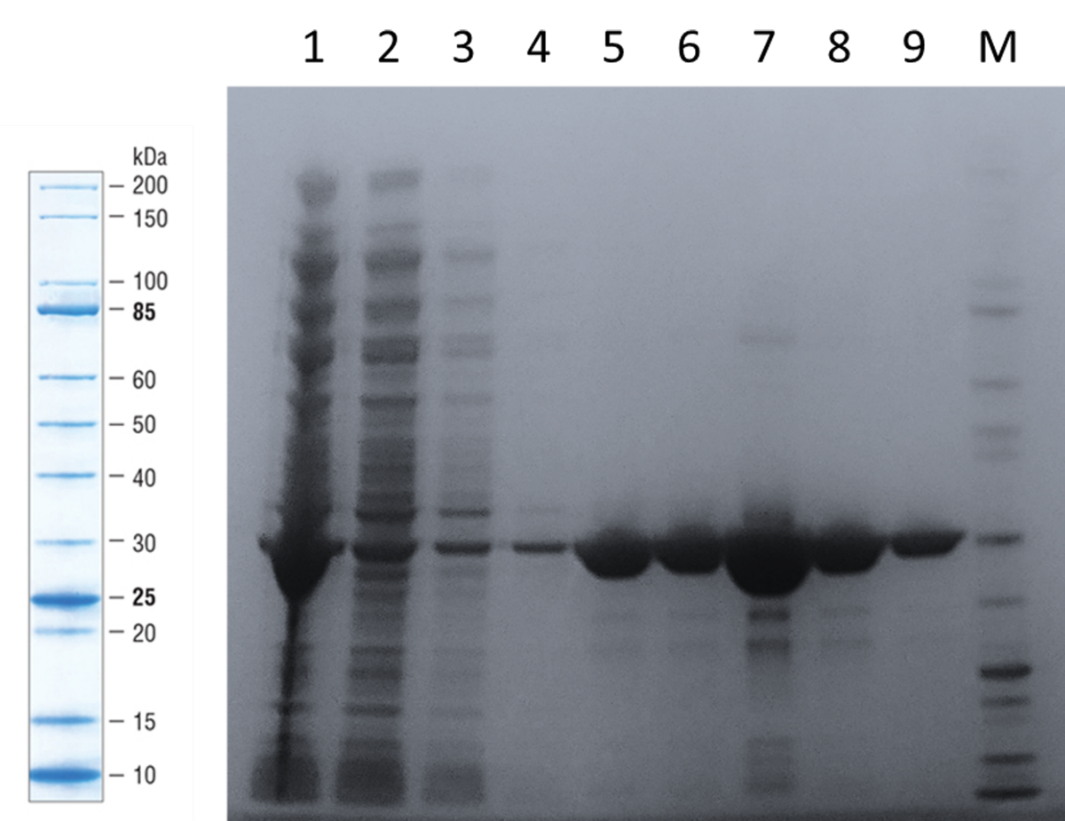
**

**Figure S6.** SDS-PAGE of His-tag affinity chromatography purification of SA01_53. Fractions: Lysate (1), Flow-through (2), Washing 1 (3), Washing 2 (4), Washing 3 (5), Washing 4 (6), Eluate 1 (7), Eluate 2 (8), Eluate 3 (9), Unstained Protein Standard Broad range (10-200 kDa) (NEB #P7704) (M).


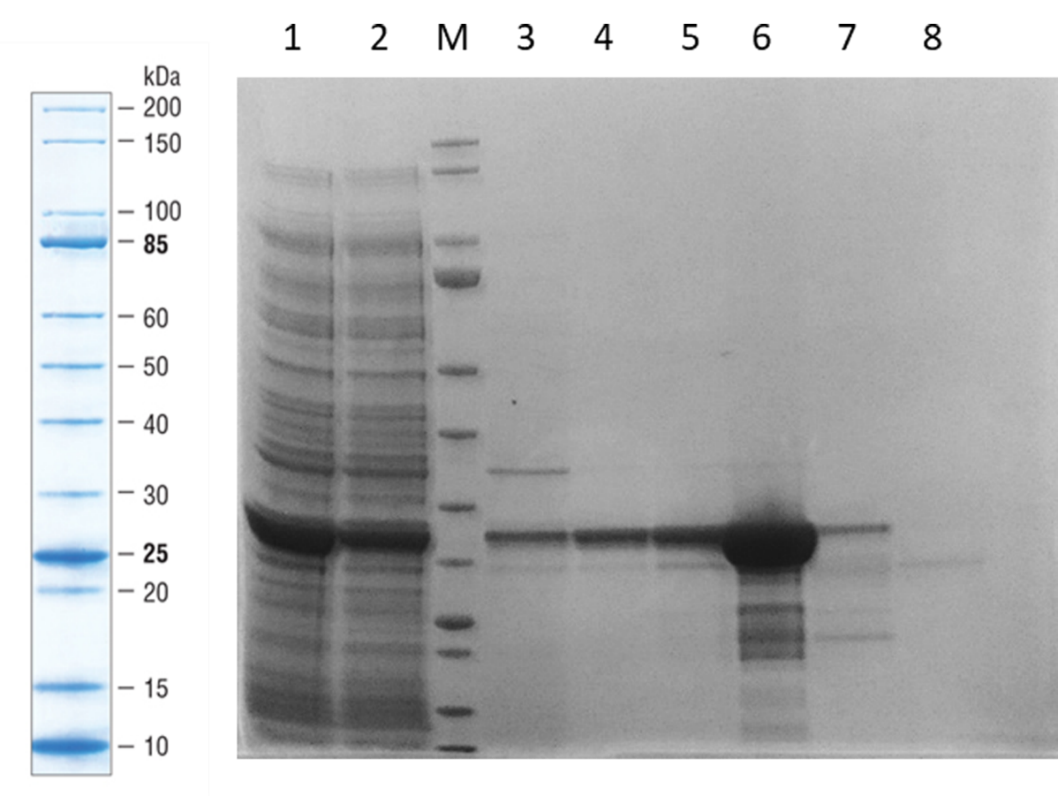


**Figure S7**. SDS-PAGE of His-tag affinity chromatography purification of recombinant 23TH_48. Fractions: Lysate (1), Flow-through (2), Washing 1 (3), Washing 2 (4), Washing 3 (5), Eluate 1 (6), Eluate 2 (7), Eluate 3 (8), Unstained Protein Standard Broad range (10-200 kDa) (NEB #P7704) (M).


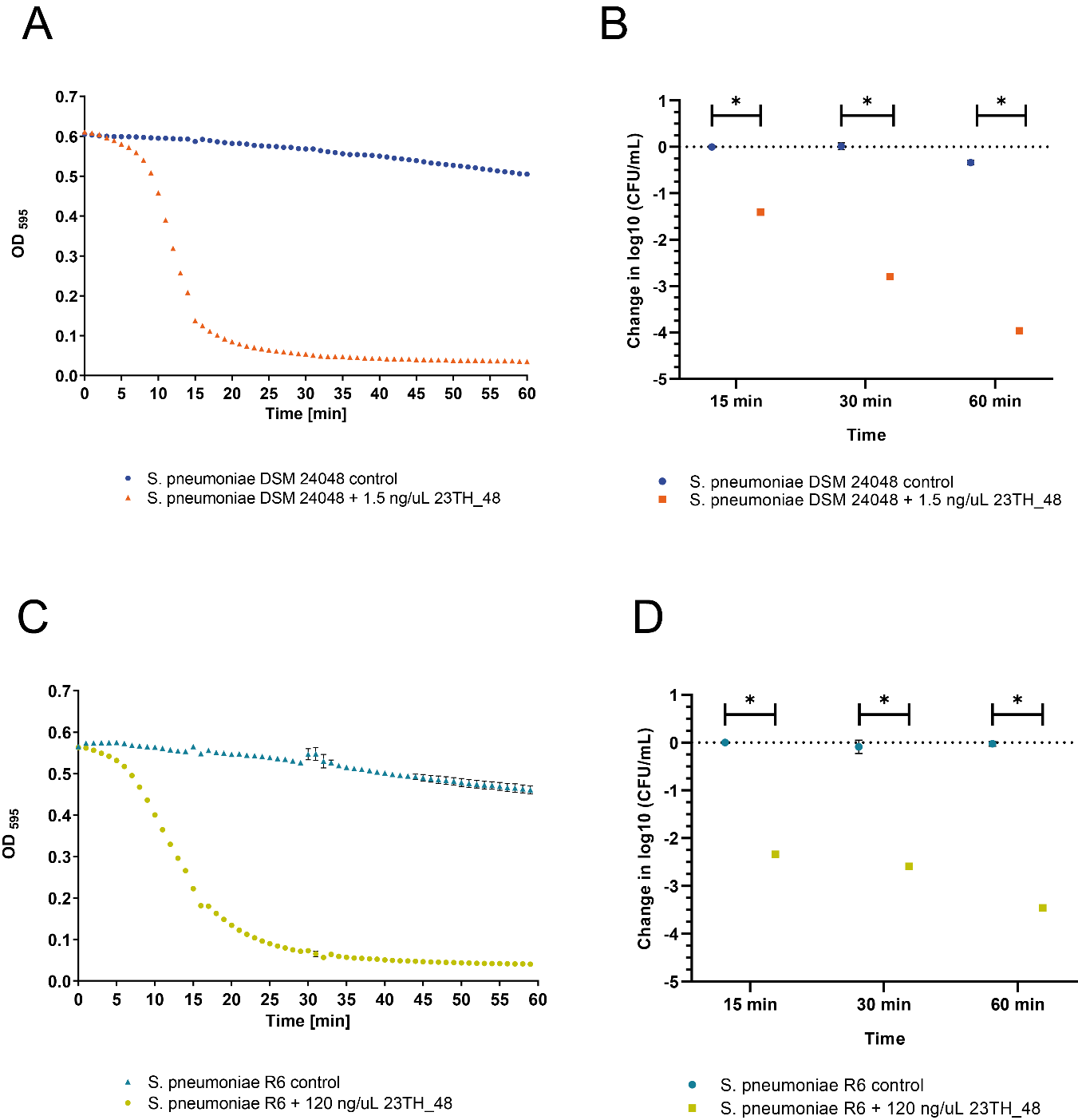


**Figure S8**. (**A**) Time kill curve of *S. pneumoniae* DSM 24048 treated with 23TH_48. (**B**) Difference of cell counts of *S. pneumoniae* DSM 24048 treated with 23TH_48. (**C**) Time kill curve of *S. pneumoniae* R6 treated with 23TH_48. (**D**) Difference of cell counts of *S. pneumoniae* R6 treated with 23TH_48.

Absorptions of time kill curves were measured at OD_595nm_ over time. For cell counts, samples were taken at 15, 30 and 60 min and cell dilutions were plated on BHI plates. Colony-forming units (CFU) were counted the next day after incubation at 37°C. Statistical analysis was done with multiple t-tests for the cell counts. Means were significantly different (*p*<0.0001)

**Table S1.** GenBank details of closely related phages to *Streptococcus* phage SA01.

| **no.** | **phage** | **accession no.** | **genome (bp)** | **GC%** | **CDS** | **tRNA*** | **DNA sequence identity (%)**** | **Shared proteins (%)***** |
| --- | --- | --- | --- | --- | --- | --- | --- | --- |
| 1 | *Streptococcus* phage SAO1 |  |  | 38 | 53 | 0 | 100 | 100 |
| 2 | *Streptococcus* phage Javan83 | MK448831.1 | 36088 | 38 | 53 | 0 | 79 | 84.91 |

*determined with ARAGORN, ** BLASTN (against SA01), ***Coregenes 3.5 (against SA01)

**Table S2**. Details of *Streptococcus* phages found to possess homology at the protein level with *Streptococcus* phage SA01.

| **no.** | **accession** | **phage** | **genome (bp)** | **GC%** | **CDS*** | **tRNA**** | **Current taxonomy - lowest two ranks (NCBI records)** |
| --- | --- | --- | --- | --- | --- | --- | --- |
| 1 | HF563658.1 | *Streptococcus* phage phiBHN167 | 37375 | 38 | 50 | 0 | *Caudovirales*; *Siphoviridae* |
| 2 | JX879087.1 | *Streptococcus* phage phiNJ2 | 37282 | 39 | 50 | 0 | *Caudovirales*; *Siphoviridae* |
| 3 | KC413988.1 | *Streptococcus* phage phiST1 | 38940 | 40 | 54 | 0 | *Caudovirales*; *Siphoviridae* |
| 4 | KT388093.1 | *Streptococcus* phage A25 | 33900 | 38 | 47 | 0 | *Caudovirales*; *Siphoviridae* |
| 5 | KX550065.1 | *Streptococcus* phage SpGS-1 | 37631 | 38 | 52 | 0 | *Caudovirales*; *Siphoviridae* |
| 6 | KY065455.1 | *Streptococcus* phage IPP14 | 37252 | 38 | 49 | 0 | *Caudovirales*; *Siphoviridae* |
| 7 | KY065479.1 | *Streptococcus* phage IPP39 | 37761 | 38 | 50 | 0 | *Caudovirales*; *Siphoviridae* |
| 8 | KY065487.1 | *Streptococcus* phage IPP48 | 37848 | 39 | 52 | 0 | *Caudovirales*; *Siphoviridae* |
| 9 | KY065490.1 | *Streptococcus* phage IPP52 | 36805 | 38 | 52 | 0 | *Caudovirales*; *Siphoviridae* |
| 10 | KY065491.1 | *Streptococcus* phage IPP53 | 37235 | 38 | 55 | 0 | *Caudovirales*; *Siphoviridae* |
| 11 | KY065493.1 | *Streptococcus* phage IPP55 | 37847 | 39 | 48 | 0 | *Caudovirales*; *Siphoviridae* |
| 12 | KY065501.1 | *Streptococcus* phage IPP65 | 39046 | 39 | 50 | 0 | *Caudovirales*; *Siphoviridae* |
| 13 | KY065502.1 | *Streptococcus* phage IPP66 | 37969 | 38 | 53 | 0 | *Caudovirales*; *Siphoviridae* |
| 14 | KY349816.1 | *Streptococcus* phage Str01 | 37030 | 38 | 52 | 0 | *Caudovirales*; *Siphoviridae* |
| 15 | MK448671.1 | *Streptococcus* phage Javan115 | 38959 | 40 | 58 | 0 | *Caudovirales*; *Siphoviridae* |
| 16 | MK448708.1 | *Streptococcus* phage Javan23 | 37964 | 35 | 59 | 0 | *Caudovirales*; *Siphoviridae* |
| 17 | MK448714.1 | *Streptococcus* phage Javan241 | 36502 | 38 | 51 | 0 | *Caudovirales*; *Siphoviridae* |
| 18 | MK448831.1 | *Streptococcus* phage Javan83 | 36493 | 38 | 55 | 0 | *Caudovirales*; *Siphoviridae* |
| 19 | MK448834.1 | *Streptococcus* phage Javan91 | 38343 | 38 | 60 | 1 | *Caudovirales*; *Siphoviridae* |
| 20 | MK448861.1 | *Streptococcus* phage Javan174 | 38137 | 42 | 59 | 0 | *Caudovirales*; *Siphoviridae* |
| 21 | MK448874.1 | *Streptococcus* phage Javan206 | 37284 | 39 | 55 | 0 | *Caudovirales*; *Siphoviridae* |
| 22 | MK448878.1 | *Streptococcus* phage Javan224 | 38781 | 38 | 62 | 0 | *Caudovirales*; *Siphoviridae* |
| 23 | MK448886.1 | *Streptococcus* phage Javan254 | 38556 | 39 | 48 | 0 | *Caudovirales*; *Siphoviridae* |
| 24 | MK448887.1 | *Streptococcus* phage Javan258 | 39028 | 38 | 60 | 0 | *Caudovirales*; *Siphoviridae* |
| 25 | MK448897.1 | *Streptococcus* phage Javan28 | 37965 | 35 | 59 | 0 | *Caudovirales*; *Siphoviridae* |
| 26 | MK448898.1 | *Streptococcus* phage Javan284 | 37997 | 39 | 57 | 0 | *Caudovirales*; *Siphoviridae* |
| 27 | MK448905.1 | *Streptococcus* phage Javan318 | 39412 | 38 | 55 | 0 | *Caudovirales*; *Siphoviridae* |
| 28 | MK448927.1 | *Streptococcus* phage Javan394 | 36484 | 34 | 53 | 0 | *Caudovirales*; *Siphoviridae* |
| 29 | MK449009.1 | *Streptococcus* phage Javan88 | 40047 | 38 | 62 | 1 | *Caudovirales*; *Siphoviridae* |

* determined with PRODIGAL, ** determined with ARAGORN

**Table S3.** GenBank details of phages closely related to *Streptococcus* phage 23TH.

| **no** | **phage** | **accession no.** | **genome (bp)** | **GC%** | **CDS** | **tRNA** | **DNA sequence identity (%)**** | **Shared proteins (%)***** |
| --- | --- | --- | --- | --- | --- | --- | --- | --- |
| 1 | *Streptococcus* phage 23TH |  | 32272 | 40 | 49 | 0 | 100 | 100 |
| 2 | *Streptococcus* phage PH10 | FN391954.1 | 31276 | 39 | 54 | 0 | 58 | 61 |
| 3 | *Streptococcus* phage Javan366 | MK448918.1 | 30862 | 40 | 50 | 0 | 55 | 68 |
| 4 | *Streptococcus* phage Javan363 | MK448739.1 | 29384 | 40 | 44 | 0 | 48 | 73 |

*determined with ARAGORN, ** BLASTN (against 23TH), ***Coregenes 3.5 (against 23TH)

**Table S4.** Details of *Streptococcus* phages found to possess homology at the protein level with *Streptococcus* phage 23TH.

| **no.** | **accession** | **Phage** | **genome (bp)** | **GC%** | **CDS*** | **tRNA**** | **Current taxonomy - lowest two ranks (NCBI records)** |
| --- | --- | --- | --- | --- | --- | --- | --- |
| 1 | FN391954.1 | *Streptococcus* phage PH10 | 31276 | 39 | 52 | 0 | *Caudovirales*; *Siphoviridae* |
| 2 | KY065453.1 | *Streptococcus* phage IPP11 | 37518 | 41 | 56 | 0 | *Caudovirales*; *Siphoviridae* |
| 3 | KY065454.1 | *Streptococcus* phage IPP12 | 34428 | 40 | 57 | 0 | *Caudovirales*; *Siphoviridae* |
| 4 | KY065458.1 | *Streptococcus* phage IPP17 | 34864 | 40 | 49 | 0 | *Caudovirales*; *Siphoviridae* |
| 5 | KY065459.1 | *Streptococcus* phage IPP18 | 34235 | 40 | 55 | 0 | *Caudovirales*; *Siphoviridae* |
| 6 | KY065460.1 | *Streptococcus* phage IPP19 | 33895 | 40 | 48 | 0 | *Caudovirales*; *Siphoviridae* |
| 7 | KY065461.1 | *Streptococcus* phage IPP20 | 37441 | 40 | 56 | 0 | *Caudovirales*; *Siphoviridae* |
| 8 | KY065462.1 | *Streptococcus* phage IPP21 | 37192 | 40 | 56 | 0 | *Caudovirales*; *Siphoviridae* |
| 9 | KY065463.1 | *Streptococcus* phage IPP22 | 36344 | 40 | 53 | 0 | *Caudovirales*; *Siphoviridae* |
| 10 | KY065469.1 | *Streptococcus* phage IPP28 | 33045 | 40 | 52 | 0 | *Caudovirales*; *Siphoviridae* |
| 11 | KY065470.1 | *Streptococcus* phage IPP29 | 33953 | 40 | 54 | 0 | *Caudovirales*; *Siphoviridae* |
| 12 | KY065471.1 | *Streptococcus* phage IPP30 | 36844 | 40 | 54 | 0 | *Caudovirales*; *Siphoviridae* |
| 13 | KY065494.1 | *Streptococcus* phage IPP57 | 33084 | 40 | 50 | 0 | *Caudovirales*; *Siphoviridae* |
| 14 | KY065495.1 | *Streptococcus* phage IPP58 | 32027 | 40 | 50 | 0 | *Caudovirales*; *Siphoviridae* |
| 15 | KY065499.1 | *Streptococcus* phage IPP63 | 34280 | 40 | 52 | 0 | *Caudovirales*; *Siphoviridae* |
| 16 | MK448732.1 | *Streptococcus* phage Javan331 | 35176 | 40 | 58 | 0 | *Caudovirales*; *Siphoviridae* |
| 17 | MK448739.1 | *Streptococcus* phage Javan363 | 29384 | 40 | 47 | 0 | *Caudovirales*; *Siphoviridae* |
| 18 | MK448830.1 | *Streptococcus* phage Javan73 | 32459 | 38 | 54 | 0 | *Caudovirales*; *Siphoviridae* |
| 19 | MK448842.1 | *Streptococcus* phage Javan110 | 31374 | 37 | 50 | 0 | *Caudovirales*; *Siphoviridae* |
| 20 | MK448894.1 | *Streptococcus* phage Javan272 | 31070 | 38 | 51 | 0 | *Caudovirales*; *Siphoviridae* |
| 21 | MK448901.1 | *Streptococcus* phage Javan306 | 32976 | 40 | 56 | 0 | *Caudovirales*; *Siphoviridae* |
| 22 | MK448912.1 | *Streptococcus* phage Javan340 | 31480 | 40 | 53 | 0 | *Caudovirales*; *Siphoviridae* |
| 23 | MK448918.1 | *Streptococcus* phage Javan366 | 30862 | 40 | 48 | 0 | *Caudovirales*; *Siphoviridae* |
| 24 | MK448920.1 | *Streptococcus* phage Javan372 | 32918 | 39 | 57 | 1 | *Caudovirales*; *Siphoviridae* |

**Table S7.** Bacterial strains used in this study.

| **Bacteria** | **Strain** | **Origin** | **Culture collection** |
| --- | --- | --- | --- |
| *Streptococcus agalactiae* | COH31rs |  | APC |
|  | Group B |  | APC |
| *Streptococcus pneumoniae* | DSM 11865 | Clinical isolate, serotype 9V | DSMZ |
|  | DSM 14377 | Serotype 3 | DSMZ |
|  | DSM 25971 | Child (7 years) with a myelodysplastic disorder | DSMZ |
|  | DSM 24048 | Human sputum, Serotype 19F | DSMZ |
|  | R6 |  |  |
| *Streptococcus infantis* | 23TH | Human saliva | APC |
| *Streptococcus anginosus* | SA1 | Human saliva | APC |
| *Streptococcus bovis* | i88  i135  i140  130 |  | APC |
| *Streptococcus dysgalactiae* | UCC 5003  Group C#2  43078  5244 |  | APC |
| *Streptococcus hyointestinalis* | 5,3 |  | APC |
| *Streptococcus infantarius* | BAA-102 |  | APC |
| *Streptococcus mutans* | SM1  APC119 |  | APC |
| *Streptococcus uberis* | 700407  5344  43382  U  U2  U3 |  | APC |
| *Streptococcus pyogenes* | DSM 2071  DSM 11728 |  | DSMZ  DSMZ |
| *Streptococcus salivarius* | G85  i4  i57  DPC6382 |  | APC |
| *Streptococcus sanguinis* | CCUG 59327 |  | APC |
| *Lactococcus lactis* | SMQ86 | Dairy Fermentation | APC |
| *Lactobacillus gasseri* | DSM 20243 | Human | DSMZ |
| *Lactobacillus acidophilus* | DSM 20079 | Human | DSMZ |
| *Lactobacillus crispatus* | DSM 20584 | Human eye | DSMZ |
| *Staphylococcus aureus* | RF 122 |  | APC |
| *Enterococcus faecalis* | 5152 |  | APC |
| *Bacillus cereus* | 6087 |  | APC |

Culture collections recorded as DSMZ, The Leibniz Institute DSMZ-German Collection of Microorganisms and Cell Cultures; APC, The APC Microbiome Ireland culture collection.

**Table S8.** Primers used in the study.

| Primer | Sequence 5‘ to 3‘ |
| --- | --- |
| 23THForBamHI | GCCGGGATCCGGGAATAAAAATG |
| 23THRevHindIII | ACCCCAAGCTTATGCCTGCGGT |
| SANGForBamHI | TAAGCAGGATCCGATGAATAAAAACGACTTATTCG |
| SANGRevHindIII | TAAGCAAAGCTTTACAATAAAACCGCAAGCC |
| 23THRevCDHindIII | TGAAGCTTTTAGACTTCTTCGGCAGC |
| 23THRev2CWBDHindIII | TGAAGCTTTTAATACATATATCCTTCAGCATCG |
| 23THRev4CWBDHindIII | TGAAGCTTTTAGATCGAGCCATCACG |
| T7Fwd | TAATACGACTCACTATAGGG |
| T7Rev | GCTAGTTATTGCTCAGCGG |

**Table S9**. Plasmids constructed in the study.

| Vector | Description |
| --- | --- |
| pET-28b(+) 23TH_48 | Expression vector for 23TH_48 lysin with N-terminal His-tag |
| pET-28b(+) 23TH_48 CD | Expression vector for catalytic domain (CD) of 23TH_48 lysin with N-terminal His tag |
| pET-28b(+) 23TH_48 2 CWBD | Expression vector for CD and 2 cell wall binding repeats (CWBD) of 23TH_48 lysin with N-terminal His tag |
| pET-28b(+) 23TH_48 4 CWBD | Expression vector for CD and 4 cell wall binding repeats (CWBD) of 23TH_48 lysin with N-terminal His tag |
| pET-28b(+) SA01_53 | Expression vector for SA01_53 lysin with N-terminal His-tag |
